# Spatiotemporal patterns of wheat response to *Pyrenophora tritici-repentis* in asymptomatic regions revealed by transcriptomic and X-ray fluorescence microscopy analyses

**DOI:** 10.1093/jxb/erad183

**Published:** 2023-05-18

**Authors:** Paula Moolhuijzen, Lilian M V P Sanglard, David J Paterson, Sean Gray, Karina Khambatta, Mark J Hackett, Ayalsew Zerihun, Mark R Gibberd, Fatima Naim

**Affiliations:** Centre for Crop and Disease Management, School of Molecular and Life Sciences, Curtin University, Bentley, Western Australia 6102, Australia; Centre for Crop and Disease Management, School of Molecular and Life Sciences, Curtin University, Bentley, Western Australia 6102, Australia; Australian Synchrotron, ANSTO, Clayton, Victoria 3168, Australia; Centre for Crop and Disease Management, School of Molecular and Life Sciences, Curtin University, Bentley, Western Australia 6102, Australia; School of Molecular and Life Sciences, Curtin University, Bentley, Western Australia 6102, Australia; School of Molecular and Life Sciences, Curtin University, Bentley, Western Australia 6102, Australia; Centre for Crop and Disease Management, School of Molecular and Life Sciences, Curtin University, Bentley, Western Australia 6102, Australia; Centre for Crop and Disease Management, School of Molecular and Life Sciences, Curtin University, Bentley, Western Australia 6102, Australia; Centre for Crop and Disease Management, School of Molecular and Life Sciences, Curtin University, Bentley, Western Australia 6102, Australia; Sichuan Agricultural University, China

**Keywords:** Asymptomatic infection, calcium distribution, defence response, necrotrophic fungal pathogen, *Pyrenophora tritici-repentis*, spatiotemporal transcriptomics, *Triticum aestivum*, tan spot, X-ray fluorescence microscopy

## Abstract

Pathogen attacks elicit dynamic and widespread molecular responses in plants. While our understanding of plant responses has advanced considerably, little is known of the molecular responses in the asymptomatic ‘green’ regions adjoining lesions. Here, we explore gene expression data and high-resolution elemental imaging to report the spatiotemporal changes in the asymptomatic green region of susceptible and moderately resistant wheat cultivars infected with a necrotrophic fungal pathogen, *Pyrenophora tritici-repentis*. We show, with improved spatiotemporal resolution, that calcium oscillations are modified in the susceptible cultivar, resulting in ‘frozen’ host defence signals at the mature disease stage, and silencing of the host’s recognition and defence mechanisms that would otherwise protect it from further attacks. In contrast, calcium accumulation and a heightened defence response were observed in the moderately resistant cultivar in the later stage of disease development. Furthermore, in the susceptible interaction, the asymptomatic green region was unable to recover after disease disruption. Our targeted sampling technique also enabled detection of eight previously predicted proteinaceous effectors in addition to the known ToxA effector. Collectively, our results highlight the benefits of spatially resolved molecular analysis and nutrient mapping to provide high-resolution spatiotemporal snapshots of host–pathogen interactions, paving the way for disentangling complex disease interactions in plants.

## Teaser

In a susceptible wheat cultivar infected with the pathogen *Pyrenophora tritici-repentis*, calcium oscillations are modified, resulting in ‘frozen’ host defence signals and silencing of the host’s recognition and defence mechanisms.

## Introduction

Plant fungal pathogens invade their hosts to acquire nutrients resulting in the disruption of host cellular function. The disruption is, however, not limited to the host cells directly in contact with the invading fungal pathogen. Necrotrophic pathogens produce and secrete necrotrophic effectors (NEs) to modulate the host defence response and kill cells on which to feed (Faris and Friesen, 2020), while the host defence responses to necrotrophs are complex and driven by the diverse mechanisms used to target host cellular processes (Wang *et al.*, 2014). Important to the molecular analysis of host–pathogen interactions is sampling of tissue. Many previous molecular analyses of diseased plant tissues do not report any specific targeted sampling, and their samples could therefore consist of a mix of symptomatic and asymptomatic regions (Andersen *et al.*, 2021). The exception is the spatially resolved transcriptome analysis of lettuce infected with *Rhizoctonia solani* (Verwaaijen *et al.*, 2019) and spatiotemporal transcriptome analysis of Arabidopsis leaves infiltrated with *Pseudomonas syringae* effector (Salguero-Linares *et al.*, 2022). The lack of spatially resolved molecular analyses of crop diseases combined with complex crop plant genomes, further confounds the accurate analysis of key molecular mechanism pertaining to susceptibility and resistance. Although, we have well-established techniques to visualize the movement of a fungal pathogen within plant tissues (Lightfoot and Able, 2010), we lack the spatially resolved molecular information due to lack of either well-developed methods or their adoption in crop plants (Hurgobin and Lewsey, 2022).

We recently reported spatiotemporal redistribution of mineral nutrients using synchrotron X-ray fluorescence microscopy (XFM) in wheat leaves infected with a necrotrophic fungal pathogen, *Pyrenophora tritici-repentis* ((Died.) Drechs.) (abbreviated to Ptr) (Naim *et al.*, 2021). However, techniques for generating spatially resolved molecular analyses or profiles, which are well-established in human medicine, are not yet optimized for crop plant tissues. A recent barley seed germination study utilized tissue specific sectioning for gene expression analysis (Liew *et al.*, 2020). In our work, to gain a more controlled insight into molecular manipulation of wheat by Ptr, with some level of spatial resolution, we applied targeted tissue sampling in the asymptomatic green region (AGR) above and below diseased symptomatic regions.

Ptr is the causal agent of wheat (*Triticum aestivum* L.) tan (or yellow) spot and characterized as a specialist necrotroph, where inverse gene for gene interaction leads to plant-induced cell death (Tan *et al.*, 2010). In susceptible wheat genotypes, Ptr produces two distinct leaf symptoms, necrosis and chlorosis (Lamari and Bernier, 1989a), driven by the interaction of secreted NEs that are recognized by corresponding dominant host sensitivity genes, *Tsn1* for Ptr ToxA, *Tsc2* for Ptr ToxB, and *Tsc1* for Ptr ToxC, and other as yet uncharacterized NEs (Ali *et al.*, 2010; Ciuffetti *et al.*, 2010; Faris *et al.*, 2013; See *et al.*, 2018; Rawlinson *et al.*, 2019). Although the production of NEs contributes to the pathogenicity of Ptr, there are many other characterized and uncharacterized host–pathogen interactions involved in the success of Ptr infection (Liu *et al.*, 2006). Ptr penetrates wheat epidermal cells within 24 h (Lamari and Bernier, 1989b; Freeman and Beattie, 2008), accomplished by the secretion of plant cell wall degrading enzymes (Oliver and Osbourn, 1995; Ciuffetti *et al.*, 2014). Differences between susceptible and resistant host reactions are evident by 72 h post-inoculation when Ptr mesophyll intercellular growth has occurred (Lamari and Bernier, 1989b; Dushnicky *et al.*, 2009). In a susceptible interaction, mesophyll cells die, and hyphae growth continues, which lead to symptom development and pathogen sporulation (Ciuffetti *et al.*, 2014).

Ptr infection results in major redistribution of macro- and micronutrients in the symptomatic regions and the AGRs surrounding the lesion in *Tsn1*-containing wheat genotypes (Khambatta *et al.*, 2021; Naim *et al.*, 2021). For necrotrophs, it is reported that the pathogen disperses a wave of toxins ahead of the pathogen invasion front (Ephrath *et al.*, 1989; Friesen and Faris, 2021), resulting in sub-optimal photosynthetic capacity of the AGR (Verwaaijen *et al.*, 2019). Bastiaans (1991) was the first to estimate the impact of fungal pathogens on the AGR of various pathosystems and referred to it as the ‘beta zone’. Bastiaans’s estimates were based on photosynthetic capacity of the system, and he concluded that the impact on the AGR was pathosystem dependent. For example, the beta zone in rice leaves infected with *Pyricularia oryzae* (a hemibiotroph) was significantly larger than that in wheat leaves infected with powdery mildew (an obligate biotroph) (Bastiaans, 1991). Consequently, the disruption of photosynthesis in the AGR may reduce plant functionality and negatively impacting crop yield (Whelan and Gaunt, 1990; Serrago, 2009).

Tan spot is a major disease of wheat, and although moderately resistant wheat cultivars and fungicide treatments can reduce disease severity, disease becomes prevalent under favourable conditions. To date, the molecular interactions resulting from Ptr infection have not been analysed in the AGR, and given the depletion of key mineral nutrients (Naim *et al.*, 2021), we sought to investigate the difference in the AGRs of two commercial wheat cultivars. To this end, we generated high-resolution nutrient maps for infected leaf sections and whole genome RNA-sequencing for 2 mm sections of the AGR, apical and basal to necrotic lesions, to probe molecular interactions between wheat and Ptr in tissues adjacent to the symptomatic region. For this, we used two wheat cultivars rated susceptible to very susceptible (SVS) (cv Scout, possessing the *Tsn1* sensitivity gene to the Ptr NE ToxA) and moderately resistant (MR) (cv Magenta, possessing the *tsn1* insensitivity gene to ToxA), which were challenged with Ptr to compare nutrient redistribution and molecular changes from an early phase to mature phase of disease. The AGR gene expression data collected from the early and mature stages of disease development were then probed for defence-related responses in the SVS and MR cultivars. We also show the impact of fungicide treatment on the recovery and functionality of the AGR in the SVS cultivar. Here, for the first time, this study presents molecular and nutrient changes that occur in the AGR of wheat leaves in response to a major necrotrophic fungal pathogen in wheat along with the tissue response to post-fungicide treatment.

## Materials and methods

### Plant and fungal material and inoculation

Spring wheat (*Triticum aestivum* L. cvs Scout and Magenta) was used in this study. The two cultivars, Scout and Magenta, are rated susceptible to very susceptible (SVS) and moderately resistant (MR), respectively (See *et al.*, 2018). Seeds were sown in potting mix (Richgro, research custom mix consisting of 50% fine composted pinebark, 20% coco peat, 30% river sand, Osmoform NXT 2 kg m^−3^, dolomite lime 2 kg m^−3^, fine lime 1 kg m^−3^, gypsum 1 kg m^−3^, iron sulphate 0.8 kg m^−3^, iron chelate 0.2 kg m^−3^) in 1.1-litre pots and maintained in a glasshouse on the Curtin University Bentley Campus between January and November 2020. Each pot contained one plant and a single fully expanded leaf during Zadoks growth stages GS33–GS37 (the top two leaves down from the flag leaf) was treated.

The Ptr race 1 isolate M4 collected from Meckering, Western Australia, in 2009 was used for this study and cultured essentially as described by Moffat *et al.* (2014) and Naim *et al.* (2021). The conidia concentration was manipulated with ultrapure water to obtain a concentration of approximately 3000 conidia ml^−1^. The commonly used protocol to isolate Ptr spores from vegetative growth includes minor mycelial fragments in the fungal inoculum. A single mature and fully expanded leaf of each plant was attached to a flat platform using double-sided tape and inoculated in the glasshouse. Droplets containing 10 µl of water or fungal inoculum were placed slightly off-centre of the leaf, 12 cm from the leaf tip. Following inoculation, the glasshouse humidity was maintained above 95% for 48 h using a fitted misting system (Fog Extra, Idrobase, Italy). A set of Scout leaves were treated with fungicide at the first appearance of symptoms (3 days post-inoculation (dpi)). For RNA sequencing, three biological replicates each consisting of a pool of four leaves (each leaf contributing 2 × 2 mm leaf disc apical and basal of symptomatic region) were harvested for each cultivar, inoculum, treatment, and time point. All 42 samples were snap-frozen in liquid nitrogen immediately after harvest and stored at −80 °C prior to RNA extraction.

### X-ray elemental mapping

To examine the *in situ* distribution of mineral nutrients in infected wheat leaves, we generated high resolution elemental maps using the XFM beamline at the Australian Synchrotron as previously described (Paterson *et al.*, 2011; Howard *et al.*, 2020; Naim *et al.*, 2021). Wheat leaf samples harvested at 3 and 8 dpi were mounted onto the single aperture Perspex mount and analysed as described in Naim *et al.* (2021). A total of five replicates for inoculated and control leaf sections were scanned for the two cultivars, at two time points. High resolution 2 μm and 5 μm elemental maps were generated for representative inoculated and control leaf sections, respectively, for each cultivar at each time point. The remainder of the replicates were imaged at lower resolution (10 μm). CSIRO’s GeoPIXE software was used to generate true-elemental images, and fluorescence emission data were converted into quantitative elemental maps as previously described (Ryan, 2000). The semi-quantitative element maps were exported from GeoPIXE as tiff files and visualized using ImageJ (Schindelin *et al.*, 2015). A detailed description of the set-up and methodology has been given previously (Naim *et al.*, 2021).

### Fungicide application

Prosaro® 420 SC Foliar Fungicide (active ingredients prothioconazole 210 g l^−1^ and tebuconazole 210 g l^−1^, Bayer Crop Science Australia), the recommended fungicide for control of tan spot, was applied at different concentrations: 100% (3 ml l^−1^), 50% (1.5 ml l^−1^), and 25% (0.75 ml l^−1^) of the prescribed label rate of 300 ml ha^−1^. Fungicide was diluted in ultrapure water and placed in a spray bottle, and plants were sprayed until the leaves were dripping. Initially, the concentration of fungicide was optimized to eliminate any adverse effects of fungicide alone. For this purpose, Prosaro was diluted (0, 25%, 50%, and 100% of the label rate) and the health of leaves monitored over 2 weeks. Leaves sprayed with 50% and 100% dilution of the fungicide showed yellowing, and all dilutions were effective in suppressing disease (Supplementary Fig. S1). Therefore, a concentration of 25% was deemed suitable for the experiments conducted for this study.

### RNA extraction, DNase treatment, RNA library preparation, and sequencing

A pool of eight wheat leaf discs from four leaves (two discs apical and basal to the symptomatic region) were harvested using a 2.0 mm biopsy punch and snap-frozen in liquid nitrogen. Leaf samples were ground to a fine powder for 30 s at 1500 r.p.m. using a 1600 MiniG tissue homogenizer. One biological replicate of treatment group V (Scout, inoculated with Ptr and treated with fungicide at 8 dpi) was lost during tissue homogenization. RNA was extracted from 41 samples using Plant/Fungi Total RNA Purification Kit (Norgen Biotek) as per the manufacturer’s instructions with on-column DNaseI treatment (15 µl of DNase I with 100 µl Enzyme Incubation Buffer was added to the column and incubated at 25 °C in the water bath for 20 minutes). RNA was eluted in 20 μl of Elution Solution A and concentrations determined using QuantiFluor RNA System (Promega) followed by storage at −80 °C. A total of 1 μg of RNA was used for library preparation using the KAPA mRNA HyperPrep Kit and the xGen UDI-UMI Adapters (Integrated DNA Technologies). The libraries were sequenced by Genomics WA (Perth, Western Australia) on the S1 lane of a NovaSeq PE150 with the aim of 40 million paired-end reads per sample. The sequence data have been deposited in the National Centre for Biotechnology Information (NCBI) sequence read archive (SRA) under BioProject PRJNA798111 accession numbers SRR17648775–SRR17648938.

### Experimental design

A randomized block design was generated with DiGGer v1.05 package in RStudio (Coombes, 2008) (Supplementary Dataset S1).

### Whole genome RNA-sequencing analysis

RNA-sequencing reads were quality checked with FastQC (Andrews, 2011) and adapters trimmed using CutAdapt (Martin, 2011). The annotated sequence genome of the hexaploid bread wheat (*Triticum aestivum*) IWGS V2.0 (International Wheat Genome Sequencing Consortium, 2014) of cultivar Chinese Spring was used as the host reference. A total of 2.4 billion RNA reads from 41 samples were aligned to the IWGS V2 reference genome sequence of wheat cv Chinese Spring. A principal component analysis of gene expression for all samples showed 44% variation was due to the cultivar and 28% due to infection by Ptr (Supplementary Fig. S2). Over 120 000 annotated high-confidence IWGS genes (International Wheat Genome Sequencing Consortium, 2014) were examined for significant wheat gene expression during Ptr infection. Minimal invasion by Ptr into the AGR was expected, and to confirm this the RNA-sequenced reads were also aligned to the Ptr isolate M4 genome (Moolhuijzen *et al.*, 2018).

Stranded paired-end (PE) reads were aligned to the reference genomes using the RNA-seq aligner Star version 2.7.0e (Dobin *et al.*, 2013) with quantMode equal to GeneCounts. Gene expression counts were calculated and guided by wheat and Ptr reference gene annotations v48. Normalization and gene dispersion estimates were performed using R v3.3.3 package DESeq2 version 1.12.4 (Love *et al.*, 2014). Significant wheat differential gene expression was set at log_2_-fold change ≥2 and a Benjamini–Hochberg adjusted *P*-value (false discovery rate) at ≤0.005 (Benjamini and Hochberg, 1995) based on contrasts. Gene plots were constructed using R v3.5.1 with chromPlot v1.10.0, GenomicFeatures v1.34.8, tidyverse v1.3.0, ggpubr v0.2.4, and ggplot2 v3.2.1 libraries. A principal component analysis of all sample gene expression was conducted using R package stats v4.1.1 (prcomp and plotPCA) and ggplot2 v3.2.1 libraries on DESeq2 variance stabilizing transformation of the count data (Supplementary Fig. S3). The analysis and data are available in an RStudio v1.3.1093 (RStudio Team, 2020) markdown notebook https://github.com/ccdmb/wheat-agr-tanspot.

### Functional analysis

Functional enrichment for differentially expressed wheat genes was determined using R version 3.5.1, topGO version 2.34.0, and Rgraphviz version 2.26.0. The set of significantly expressed gene ontologies (GO) were compared with the ontologies for the genome’s total number of genes (population background) to determine enrichment using classic Fisher’s exact test. GO terms were selected as enriched with *P*-values ≤0.05. Bar plots were constructed using R v3.5.1, ggpubr v0.2.4, and ggplot2 v3.2.1 libraries. The closer examination of significantly differentially expressed genes (SDEGs) was conducted using AgriGO2 (Tian *et al.*, 2017) and biomaRt v2.52.0 using Ensembl Plants Genes 53, ‘taestivum_eg_gene’ data.

### Cluster analysis of significantly differentially expressed genes

The normalized wheat gene expression data (log_2_) from DESeq2 was filtered for the identified SDEGs and were clustered using an optimized consensus clustering tool Clust v1.17.0 (Abu-Jamous and Kelly, 2018) using a BiCoPaM method for seed cluster production and the M-N scatter plot technique for cluster evaluation and selection to extract optimal co-expressed gene clusters.

### Sequence analysis

Protein sequences were retrieved from EnsemblPlants BioMart genomes release 48 (Smedley *et al.*, 2015). Wheat gene homeologue and paralogue searches were conducted through EnsemblPlants (Howe *et al.*, 2020), and protein domain searches were conducted using InterProScan (Quevillon *et al.*, 2005; Mitchell *et al.*, 2019).

## Results

### Ptr infection elicits differential redistribution of potassium, calcium, and manganese in susceptible and moderately resistant wheat cultivars

**Fig. 1.**
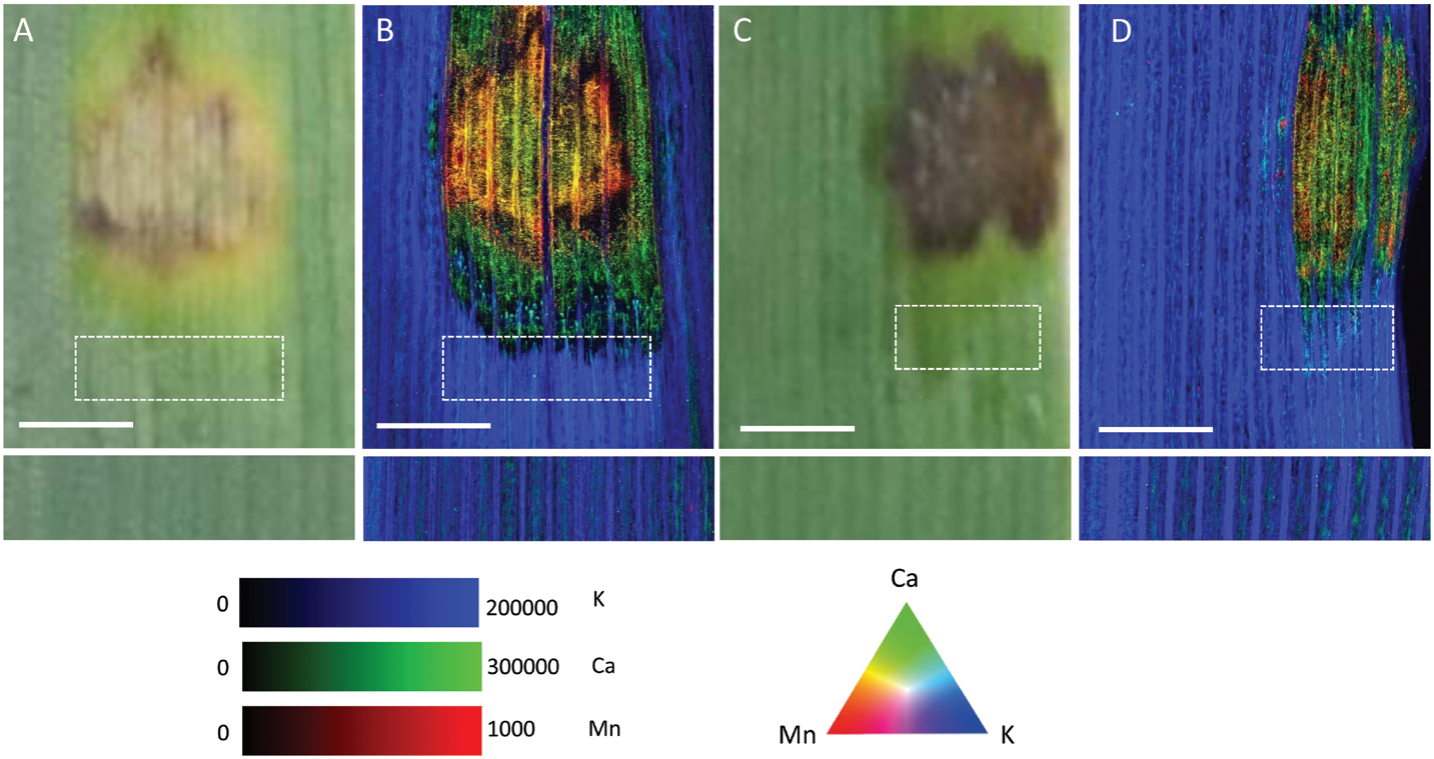
XFM-generated K, Ca, and Mn elemental maps show similarities and contrasting redistribution of elements in response to Ptr infection. High-resolution elemental maps generated for representative wheat leaves infected with Ptr and harvested 8 d post-inoculation and imaged. (A, C) Light images of the region of the leaf imaged with XFM for susceptible to very susceptible (SVS) (A) and moderately resistant (MR) (C) cultivars. (B, D) The corresponding tricolour overlay of elemental maps for Mn (red), Ca (green), and K (blue) shown for SVS (B) and MR (D) cultivars. Mn is hyper-accumulated in the centre of the symptomatic region whereas Ca is hyper-accumulated, and K is depleted, in the symptomatic region. The AGR apical to the symptomatic region is indicated by the white dashed rectangle. Each map is false-coloured to help visualize the patterns of elemental redistribution, and brightness and contrast were kept consistent for each element. Scale bar: 1 mm; elemental maps are expressed in units of ng cm^−2^.

Previously, using XFM we showed that Ptr infection resulted in major redistribution of mineral nutrients in the symptomatic and the asymptomatic green regions of a susceptible wheat cultivar (Naim *et al.*, 2021). In this study, we compared the impact of Ptr infection on two wheat varieties rated SVS and MR to tan spot, at early (3 dpi) and later (8 dpi) stages of disease development. While the effect of Ptr infection on chemical element distribution was unremarkable at 3 dpi (Supplementary Fig. S4), by 8 dpi the element maps revealed hyper-accumulation of manganese (Mn) and depletion of potassium (K) in the symptomatic region of the SVS and MR wheat, which were more pronounced in the SVS wheat. By contrast, Mn accumulation was consistent in the symptomatic region in both wheat cultivars. A distinct pattern of accumulation and depletion for calcium (Ca) was observed in the symptomatic region of the SVS cultivar, whereas in the MR cultivar Ca accumulated in both the symptomatic tissue and the AGR (Fig. 1).

**Fig. 2.**
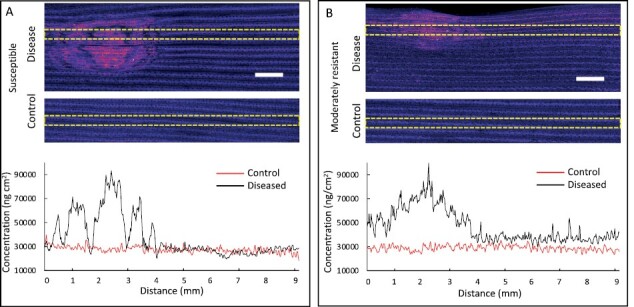
Semi-quantitative Ca images reveal differences in redistribution of Ca in the symptomatic and asymptomatic green region of SVS and MR wheat infected with Ptr. XFM Ca maps for susceptible to very susceptible (SVS) (A) and moderately resistant (MR) (B) wheat infected with Ptr and harvested 8 d post-inoculation. The Ca maps are false-coloured to help visualize the patterns of elemental redistribution, and brightness and contrast were kept consistent for the two cultivars. The concentration of Ca is measured across the leaf indicated by the yellow dashed regions; upper panel is infected leaf section and lower panel is paired control leaf section. The corresponding Ca concentration plots show a large increase in the symptomatic region with levels returning to control levels for the asymptomatic region for the SVS cultivar, whereas the MR cultivar shows increase in Ca within the imaged 5 mm region of AGR, apical to the symptomatic region. The accumulated Ca is surrounded by halos of depletion in the SVS wheat leaf section. Scale bar: 1 mm; elemental maps are expressed in units of ng cm^−2^.

**Fig. 3.**
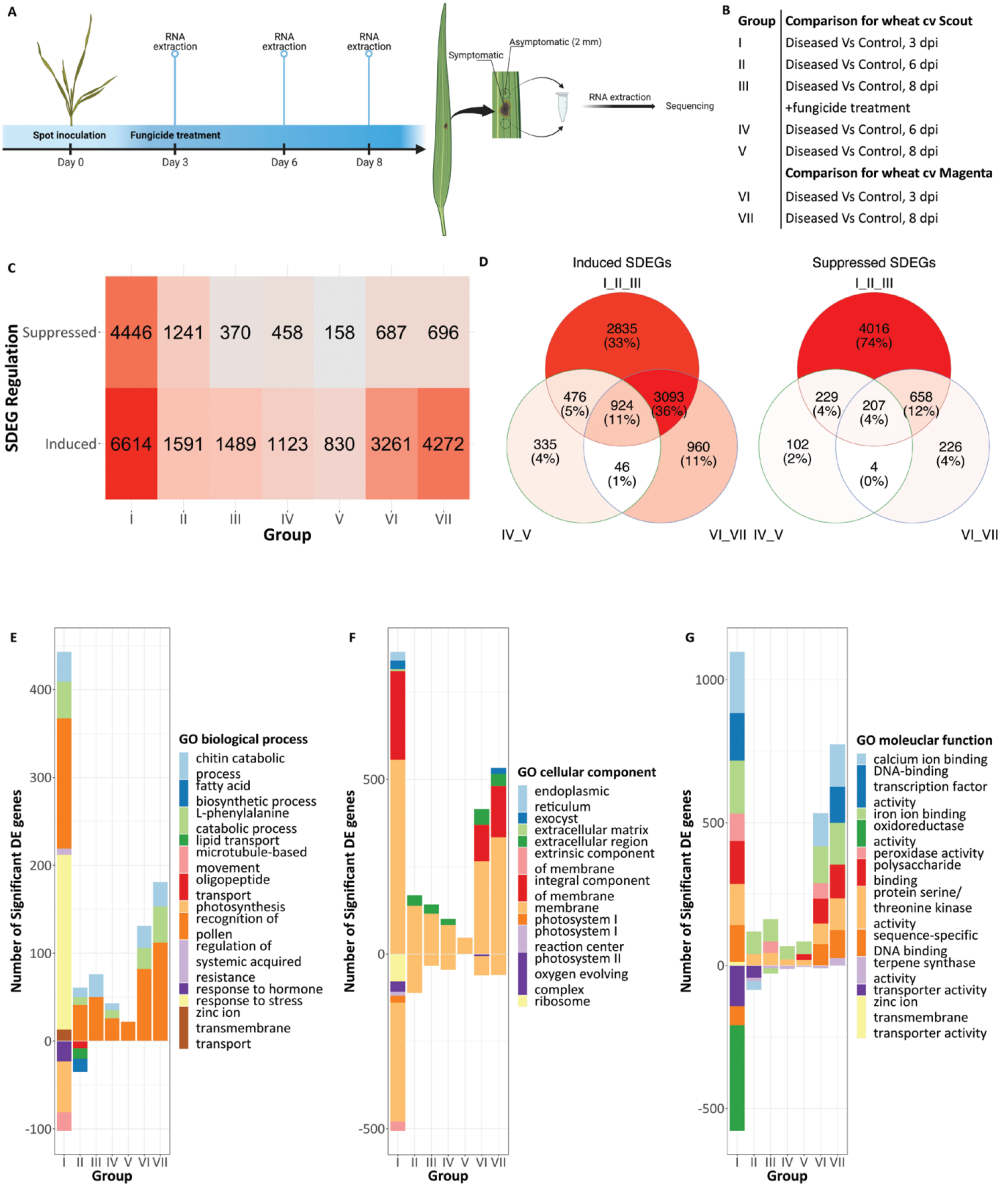
Gene expression analysis of wheat leaves infected with *Pyrenophora tritici-repentis*. (A) Timeline of the experimental set-up and overview of 2 mm disc harvested from the asymptomatic green region for RNA sequencing. Created with BioRender.com. (B) Summary of treatment groups analysed for gene expression changes. (C) Summary of number of significantly differentially expressed genes (SDEGs) for each treatment group. SDEGs were identified at an adjusted *P*-value ≤0.005 and an absolute log_2_ fold change in expression ≥2. (D). Venn diagrams showing unique and overlapping genes up- and/or down-regulated for SVS wheat with no fungicide (groups I, II, and III) or with fungicide (groups IV and V) and for MR wheat (groups VI and VII) during Ptr infection shown in (B). (E–G) Gene ontology (GO) terms significantly enriched (*P*-value <0.001 for visualization) by SDEGs for biological processes (E), cellular components (F), and molecular functions (G).

Calcium signalling is an important defence mechanism against biotic stresses, and the two wheat varieties showed the most contrasting pattern of Ca distribution. The semi-quantitative Ca maps showed an overall accumulation of Ca in the symptomatic region of the infected leaf in both the SVS and MR wheat (Fig. 2). However, a distinct redistribution pattern occurred in the SVS cultivar within the symptomatic region whereby Ca accumulation was surrounded by distinct halo of depleted region (Fig. 2A; Supplementary Fig. S5) compared with the pattern observed in the MR cultivar (Fig. 2B). Moreover, the Ca concentration data measured across leaf sections shows that Ca concentrations return to ‘background’ levels in the AGR of the SVS cultivar (Fig. 2A), whereas in the MR cultivar, increased Ca concentration was present in the AGR (Fig. 2B). To further elucidate the differences in the molecular responses in the AGR, we performed RNA-sequencing of 2 mm AGR leaf tissue samples taken apical and basal to the symptomatic region, and the AGR is indicated by the dashed circle in Fig. 3A.

### An early extensive asymptomatic green region gene expression response in the SVS cultivar is not sustained, in contrast to the MR cultivar, which maintained up-regulation of defence-related genes

To localize and analyse molecular interactions within the AGR, a defined size and location of leaf tissue was used. The gene expression in the AGRs for Ptr inoculated and non-inoculated (control) samples of SVS and MR wheat were analysed at 3 dpi (groups I and VI) and 8 dpi (groups III and VII), respectively (Fig. 3B)—where groups II and IV include differential gene expression analysis for SVS wheat infected and treated with or without fungicide, respectively, and sampled at 6 dpi, while group V is SVS wheat infected and treated with fungicide and sampled at 8 dpi. The comparative analysis identified 6614 and 1489 up-regulated and 4446, and 370 down-regulated SDEGs in Ptr inoculated SVS cultivar samples relative to the control samples at 3 and 8 dpi, respectively (Fig. 3C; Supplementary Dataset S2). The largest change in gene expression (11 060 SDEGs) was observed during the early phase of infection at 3 dpi in the SVS cultivar and greatly reduced (1859 SDEGs) at 8 dpi (Fig. 3C, D). In the MR cultivar a total of 3261 and 4272 SDEGs were up-regulated and 687 and 696 down-regulated at 3 dpi (group VI) and 8 dpi (group VII), respectively. In contrast to the SVS cultivar, the MR wheat had an increase in number of SDEGs at 8 dpi as compared with 3 dpi (Fig. 3C, D). Overall, 924 (11%) up-regulated and 207 (4%) down-regulated SDEGs were common to both cultivars across all groups (Fig. 3D).

### GO term analysis shows significant perturbations of molecular regulation at 3 dpi in the SVS compared with the MR cultivar

For the SVS cultivar, analysis of GO terms enriched by SDEGs showed that the highly induced gene expression at 3 dpi was for response to stress, regulation of systemic acquired resistance, calcium ion binding, zinc ion transmembrane transport, and DNA-binding transcription (Fig. 3E, F). These were absent at the later time points (6 and 8 dpi). Similarly, the down-regulated SDEGs at 3 dpi were related to photosynthesis, oxidoreductase activity, sequence-specific DNA binding, and response to hormone, and they were absent by 8 dpi (Fig. 3E, F; Supplementary Dataset S3). By contrast, in the MR cultivar, these GO terms enriched by down-regulated SDEGs were absent even at 3 dpi. These results show that in SVS wheat, gene expression in the AGR is highly disturbed during the early stages of disease development, which is mostly negated by 8 dpi. In contrast, the number of genes regulated in the MR cultivar increased at 8 dpi.

### Ptr driven disruptive gene expression observed in the asymptomatic green region of SVS wheat during early disease phase is associated with necrosis

**Fig. 4.**
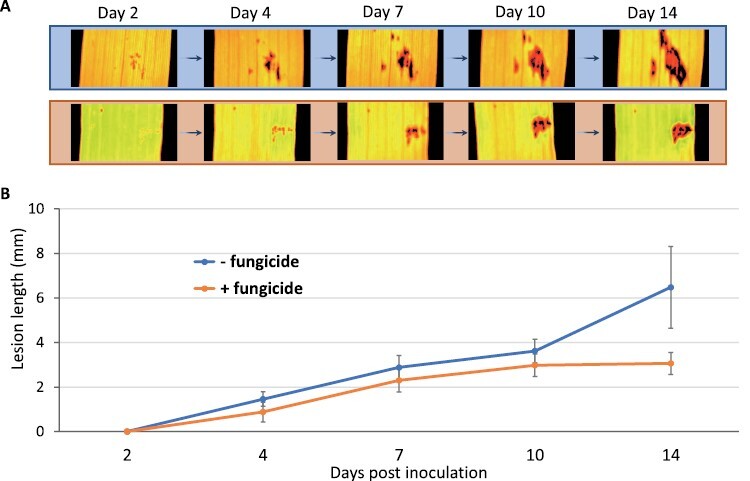
Disease progression after disease disruption in susceptible to very susceptible (SVS) wheat inoculated with Ptr and treated with fungicide. (A) Chlorophyll fluorescence images of dark adapted representative susceptible to very susceptible (SVS) wheat leaves showing lesion development over 14 days post-inoculation (dpi) with Ptr and treated with or without fungicide. Inoculated leaves were treated with fungicide at 2 dpi and lesion length imaged and measured at 2, 4, 7, 10, and 14 dpi. (B) Corresponding plot of lesion length measured from RGB images of leaves. The plot and the images show that the lesion continues to expand in untreated leaves beyond 10 dpi. Error bars are standard error of mean (*n* = 5).

To understand association of Ptr driven disruptive gene expression observed during the early phase of the disease in the SVS cultivar, the inoculated SVS wheat leaves were treated with fungicide as the disease symptoms started to appear. The lesion length was monitored over a 2-week period (Fig. 4). The lesion in the fungicide-treated leaves extended to a final length of 2.98 ± 0.51 mm by 10 dpi. By contrast, the lesion in the untreated leaves continued to extend reaching 6.48 ± 1.83 mm by the end of the observation period of this study at 14 dpi (Fig. 4). This suggests that the AGR amounting to ~50% of the symptomatic region failed to recover after disease disruption with fungicide application. The gene expression analysis showed that the disruptive levels of gene regulation settled down by 8 dpi (Fig. 3), but the lesion continued to extend.

The role of fungicide treatment on recovery of molecular processes was subsequently examined. Ptr inoculated and control leaves were treated with fungicide at the onset of disease symptoms (3 dpi) and leaf samples were harvested at 6 dpi (group IV) and 8 dpi (group V) for differential gene expression analysis (Fig. 3B). The comparative analysis of control and diseased leaves treated with fungicide (groups IV and V) to the untreated (groups II and III) in the SVS cultivar revealed substantial reductions in the number of SDEGs amounting to 30–45% and 57–63% reductions in induced and suppressed genes, respectively (Fig. 3C). The total number of SDEGs up-regulated and down-regulated were 1123 and 458 at 6 dpi and 830 and 158 at 8 dpi, respectively (Fig. 3C and Supplementary Dataset S2). The GO terms not found enriched by SDEGs in no fungicide treatment (group II) as compared with fungicide treatment (group IV) were fatty acid biosynthesis, lipid transport, oligopeptide transport, transporter activity, and calcium ion binding at 6 dpi, and peroxidase activity was absent at 8 dpi (Fig. 3E, F). These results indicate that although fungicide treatment is unable to recover the initially compromised AGR, it stops the pathogen from causing further damage.

### Moderately resistant wheat asymptomatic green region has a prolonged defence activation

From the nutrient maps, a key difference between the SVS and MR wheat was the increased concentration of Ca in the AGR of MR (Fig. 2). Consistent with this observation, the GO terms showed sustained and higher expression of SDEGs involved in Ca ion binding at 3 and 8 dpi, respectively, in the MR cultivar (Fig. 3G). Although SDEGs for Ca transport were found to be up-regulated for all treatments, in the AGR the number of SDEGs between 3 and 8 dpi decreased in the SVS but increased in the MR cultivar (Fig. 3G; Supplementary Dataset S3).

**Fig. 5.**
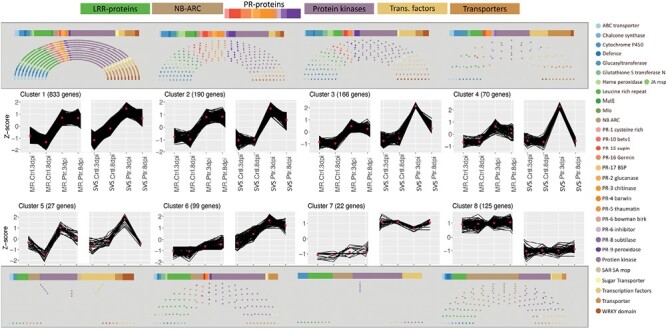
Defence-related SDEG co-expression profiles for the AGR in susceptible to very susceptible (SVS) and moderately resistant (MR) wheat inoculated with Ptr at 3 and 8 dpi. SDEGs with similar gene expression patterns are shown clustered into eight profiles. The number of genes in the cluster are coloured by defence-related annotations and plotted above and below the gene expression profiles.

To gain a better insight into genes involved in defence mechanisms, SDEGs were classified into pathogen sensing and signalling, transcription activation, hormone signalling, secondary metabolite biosynthesis, pathogenesis-related proteins, and transport. For this, 4249 defence-related SDEGs were put through gene expression cluster analysis. A total of 1532 genes clustered into eight highly correlated co-expression profiles (Fig. 5). The largest co-expression profile, cluster 1, contained over half of the clustered genes (833 genes). Protein kinases constituted the majority in all the clusters, except in clusters 5 and 8 in which transcription factors and NB-ARC proteins were dominant, respectively. Defence-related PR protein were not represented in the smaller clusters 5 and 7. The expression profile for clusters 1 and 2 showed the effect of Ptr inoculation in both cultivars, with induced expression at 3 and 8 dpi. Clusters 3–5 showed expression profiles that were highly induced at 3 dpi as compared with 8 dpi in the SVS cultivar and as compared with the MR cultivar. Clusters 7 and 8 contained cultivar-dependent genes, which were largely represented by protein kinases, NB-ARC proteins, leucine-rich repeat (LRR) proteins, and transcription factors. These were constitutively down-regulated in the SVS as compared with the MR cultivar (cluster 7) and constitutively up-regulated in the MR as compared with the SVS cultivar (cluster 8).

**Fig. 6.**
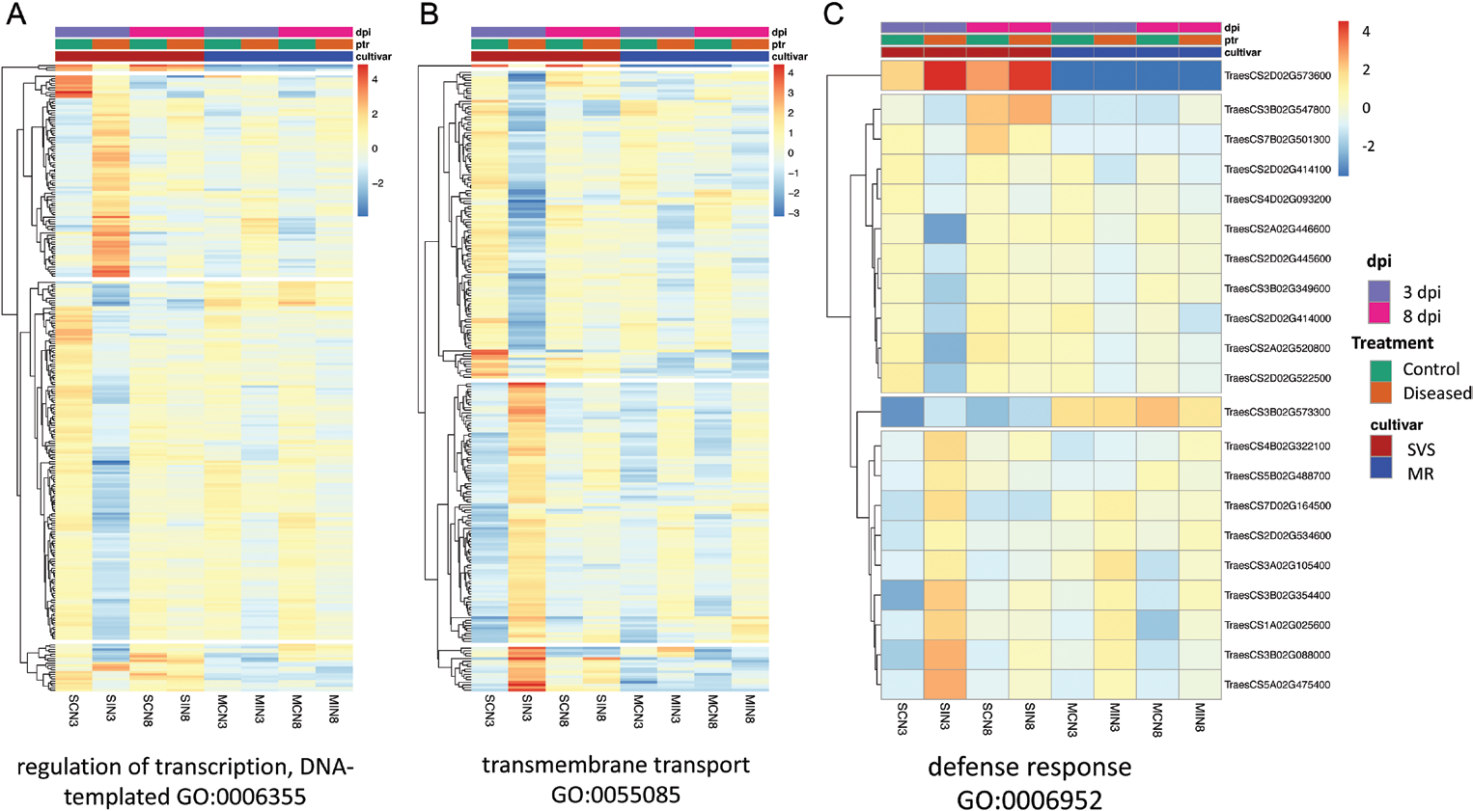
Defence-related gene expression for wheat Ptr moderately resistant (MR) and susceptible to very susceptible (SVS) cultivar control and Ptr inoculated samples not treated with fungicide at 3 and 8 dpi. SDEG expression (average log_2_–log_2_) is shown for regulation of transcription (GO:0006355) (A), transmembrane transport (GO:0055085) (B), and defence response (GO:0006952) (C).

The general expression pattern of the nucleotide binding site (NBS)–LRR domain containing proteins (NBS-LRRs) also showed that the up-regulated gene expression in the SVS cultivar at 3 dpi was not maintained at 8 dpi as compared with the moderately resistant cultivar (cluster 6). Notably, an NB-ARC-LRR domain-containing protein gene (TraesCS2D02G573600) had expression up-regulated in the SVS cultivar at 3 dpi (adjusted *P*-value 3.52 × 10^−16^) and 8 dpi and was not expressed in the MR cultivar (Fig. 6), while another NB-ARC and Rx N-terminal domain protein gene (TraesCS3B02G573300) had relatively higher expression in the MR as compared with the SVS wheat (Supplementary Fig. S6).

SDEGs involved in hormone signalling and defence against necrotrophic pathogens were also investigated. In the ethylene-activated signalling pathway, although SDEG regulation at both time points (3 and 8 dpi) was detected in the MR cultivar, supressed gene regulation in the SVS cultivar was identified at 3 dpi but not at 8 dpi (Supplementary Dataset S3). In the jasmonic acid (JA)-mediated signalling pathway, E3 ubiquitin-protein ligase gene expression was induced in the SVS cultivar only at 3 dpi, and at 3 and 8 dpi in the MR cultivar, while TZF1 was down-regulated only at 3 dpi in the SVS cultivar and at 3 and 8 dpi in the MR cultivar. Chloroplast allene oxide synthase related to JA biosynthetic processes was induced in both the SVS and MR cultivars. SDEGs for EDTS5 involved in systemic acquired resistance in the salicylic acid (SA)-mediated signalling pathways were highly induced in both the SVS and MR cultivars, but absent in the SVS cultivar at 8 dpi as compared with the MR cultivar. Furthermore, two cysteine/histidine-rich DC1 protein domain-containing genes, TraesCSU02G176200 (at 3 dpi, log_2_FC 21.25 and adjusted *P*-value 1.22 × 10^−71^) and TraesCS2B02G521300 (at 8 dpi, log_2_FC 23.66 and adjusted *P*-value 8.69 × 10^−94^), whose products positively regulate plant defence during microbial infection (Hwang *et al.*, 2014), were highly up-regulated in the MR as compared with the SVS cultivar (Supplementary Dataset S3).

### Ptr gene expression is detected in the asymptomatic green region

**Fig. 7.**
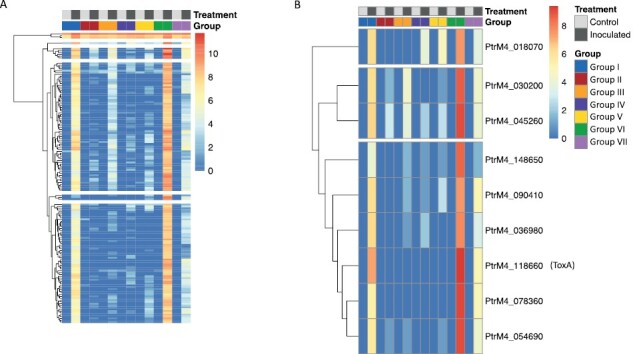
*Pyrenophora tritici*-*repentis* detection in AGR. (A) Heatmap of Ptr gene read counts in sample groups (refer to Fig. 3B) collected from wheat leaves. (B) Subset of Ptr genes predicted as putative effectors and *ToxA*.

Although our sampling was targeting analysis of the asymptomatic region, our sequencing results also captured Ptr genes. Previously, using elemental maps, we reported that the fungus hyphae extend ahead of the lesion front (Naim *et al.*, 2021) at least during the early phase of disease. A higher count of pathogen reads was found in the MR cultivar at 3 dpi, which was greater (60 000 PE reads) as compared with the SVS cultivar (18 000 PE reads). Overall, a total of 148 Ptr genes were expressed in the AGR (across all samples) although the pathogen expression levels were too low for differential analysis (Fig. 7A). Of these, 44 predicted protein sequences contained a predicted signal peptide, with nine of these predicted as effectors (two cytoplasmic and seven apoplastic), which included the known NE *ToxA* (PtrM4_118660) (Fig. 7B; Supplementary Fig. S7). Expression of ToxA and PtrM4_078360 were both detected at 3 dpi and 8 dpi in the MR and only at 3dpi in the SVS cultivar.

## Discussion

The physiological and molecular response of a tissue undergoing environmental stress is linked to, and modulated by, the individual responses of the cells that make up the tissue. In essence by spatially resolving these responses, we gain a better understanding of stress-driven intercellular communication. While our approach does not allow cellular scale probing, the spatially resolved sampling scheme we employed represents a significant step in the right direction, providing a temporal snapshot of the interaction between wheat and Ptr. Using a combination of high-resolution microscopy and whole genome RNA sequencing, molecular changes were captured in the AGR immediately above and below Ptr inoculated symptomatic regions of wheat rated SVS and MR to Ptr during early and late phase of disease. In this study, we show that in a tan spot sensitive cultivar, Ca signalling and gene regulation is controlled in a manner that supports the proposed necrotrophic hijacking of wheat defence responses (Faris and Friesen, 2020). Moreover, the Ca elemental maps suggest a tightly controlled hijack of the system where the host defence signals are encapsulated ultimately favouring further pathogen invasion.

### Ptr tightly controls defence responses in the SVS wheat enabling it to invade the host

In this study, we show for the first time that the AGR surrounding a symptomatic lesion does not recover following disease disruption by fungicide, and an early heightened host defence in the sensitive cultivar sets an irreversible condition for necrosis. The SVS cultivar AGR, 22 times the size of the lesion at the time of fungicide treatment, was lost to tan spot lesion development, indicative of the extent of spread of the NEs during the early phase of disease (Fig. 4). This was supported by substantial initial induction then suppression of the number of genes related to defence responses, which by contrast was relatively stable in the MR cultivar AGR (Fig. 3). This pattern of defence regulation was previously proposed as a pathogen-driven hijacking of the host defence (Faris and Friesen, 2020) potentially driven by interaction of Ptr NEs (ToxA and others) with host defence genes (*Tsn1* and others). This was shown for *Parastagonospora nodorum* (Pn) NE, SnTox1, which interacts with wheat sensitivity gene, *Snn1*, a plant receptor kinase with an intracellular protein kinase domain, transmembrane domain, and extracellular calcium- and galacturonide-binding domain, which triggers damage-associated molecular patterns and pathogen triggered immunity (PTI) (Shi *et al.*, 2016; Faris and Friesen, 2020). It is the recognition of pathogen secreted effectors, effector-triggered immunity, which generates a hypersensitive response and localized cell death (Ali *et al.*, 2018) that deprive the pathogen of nutrients (Mengiste, 2012). This can be effective in limiting biotrophic pathogens invasion, but conversely nourishes a necrotrophic lifestyle, and enables the pathogen to further invade and develop disease (Faris and Friesen, 2020). We speculate that in the early stages of infection the AGR is primed in the same manner for tissue damage that ultimately predisposes and compromises the region to invasion by advancing mycelia from the adjoining symptomatic region and/or secondary infections from spore germination.

The initial burst of NEs is potentially captured by the Ca redistribution pattern, specifically the central hyper-accumulation of Ca observed in the XFM maps (Fig 2). This initial increase in Ca concentration in the symptomatic region is followed by two further waves or rings of Ca accumulation that were potentially triggered by secondary and tertiary waves of Ptr NEs. Calcium signalling is an important defence mechanism against biotic stresses with rapid increases of Ca^2+^ observed in PTI (Yuan *et al.*, 2017), and our results may indeed support the suppression of PTI in the AGR allowing disease development. The plant nucleotide binding leucine-rich repeat (NLR) resistosome forms a calcium-permeable channel triggering the hypersensitive response and cell death (Bi *et al.*, 2021). The wheat genome encodes a multitude of NLRs, and although their role in wheat disease resistance is largely unknown, they are thought to monitor the status of plant proteins that are targeted by pathogen effectors (Shao *et al.*, 2019). In wheat CNL (NLRs with coiled-coiled domain) resistosomes have been shown to form a pore in the plasma membrane to induce cell death (Förderer *et al.*, 2022). In an inverse gene-for-gene disease model, the wheat *Tsn1* locus, for example, located on wheat chromosome 5BL, encodes a predicted NLR domain and a serine/threonine protein kinase that confers host sensitivity to Ptr ToxA (Faris *et al.*, 2010).

In our study, we identified cultivar specific differential expression for two NB-ARC domain-containing genes. TraesCS3B02G573300, encoding an NB-ARC and Virus X resistance protein-like coiled-coil domain containing protein with a PANTHER classification (Rawlings *et al.*, 2018) for a plant disease resistance protein (PTHR23155), had relatively higher expression in MR as compared with SVS wheat (Fig. 5). The second gene, TraesCS2D02G573600, encoding an NB-ARC, LRR and predicted coiled-coil (Rx, N-terminal) domain-containing protein, was not expressed in the MR but up-regulated in the treated SVS cultivar. As TraesCS3B02G573300 encodes a protein that lacks any LRR domain and TraesCS2D02G573600 encodes a protein that only contained an Rx, N-terminal domain, which is a predicted coiled-coil structure that adopts four helical bundle folds as opposed to five identified in the pentameric resistosome structure in wheat, these two genes may not be part of the resistosome (Förderer *et al.*, 2022). However, as the NB-ARC domain signalling motif is often found in plant resistance genes and regulators of cell death (van Ooijen *et al.*, 2008), the two differentially expressed genes identified in this study may play a role in Ptr defence and warrant further investigation.

The suppression of oxidoreductase and photosynthesis activities contributes directly to tissue damage (necrosis) (Manning *et al.*, 2013) and compromised energy production. In the ToxA-sensitive SVS cultivar, ToxA-induced protein changes in photosystem I and II lead to light-dependent accumulation of reactive oxygen species in the chloroplasts (Manning *et al.*, 2009), which is required for necrosis. In the SVS cultivar, a massive shift in the regulation of transcription factors was also evident including active suppression of σ70 RNA polymerases, which have been shown to be involved in transcription of photosynthesis-related genes (Isono *et al.*, 1997). The disruption of chloroplast metabolism may be key to the changes observed in SA and JA gene regulation.

### The asymptomatic green region of MR wheat is armed with more active defence

In this study both SVS and MR wheat cultivars had systemic acquired resistance (SAR)–SA-mediated signalling pathway genes activated in the AGR, but these were absent in the SVS cultivar at 8 dpi. As the SAR–SA-mediated signalling pathway gene *EDTS5* (encoding l-lysine α-aminotransferase) is a suppressor of Enhanced Disease Resistance 1 (EDR1), i.e. a negative regulator of defence (Ajigboye *et al.*, 2017), its absence by 8 dpi indicates a compromised defence in the SVS cultivar in the later stages of disease. It does appear that in the AGR, not only is the JA and ethylene-associated defence against necrotrophic pathogens active, but so is the ethylene-enhanced SA response normally associated with biotrophic pathogen resistance, which is proposed to suppress JA responses (Ajigboye *et al.*, 2017). Concordantly, the JA-mediated signalling pathway was only induced at the early time point for the SVS cultivar and down-regulated in the MR cultivar.

It has been shown that SAR is involved in signalling mechanisms that helps to prevent further pathogen attack and provides a long-lasting resistance to secondary infections (Conrath, 2006). An overall decrease in the number of genes involved in SAR and the ring of Ca depletion at the edge of the symptomatic region at 8 dpi in the SVS cultivar suggests an increasingly compromised position of the SVS cultivar’s AGR and desensitization to further attacks (Figs 2, 3). The gene expression data are consistent, with an induction of calcium ion binding in both SVS and MR cultivars at 3 dpi and only maintained with further up-regulation in the MR cultivar at 8 dpi. A report of lettuce leaves infected with the necrotroph *Rhizoctonia solani* AG1-IB also showed induction of calcium signalling in the AGR and further deduced that programmed cell death and activation of the hypersensitive response were occurring as localized events in the symptomatic zone (Verwaaijen *et al.*, 2019). In this case negative regulation of programmed cell death was induced in the more viable cells to potentially support the biotrophic growth phase of the pathogen.

### The implications of targeted tissue sampling for studying plant disease interactions in crop plants

The application of spatiotemporally resolved microscopy and gene expression data enabled us to capture the complexity of the Ptr–wheat interaction in the AGR that is not reported previously. Although the AGR was sampled, Ptr gene expression was detected at 3 dpi (Fig. 7). This is in consensus with our nutrient maps showing accumulation of Cu in fungal hyphae in the AGR, revealing that Ptr outgrows the symptomatic region during the early phase of the disease (Naim *et al.*, 2021). Extraction of RNA from 2 mm discs of leaf tissue narrowed the ratio of plant cells to Ptr, which enabled us to detect expression of various Ptr genes in the AGR including high expression of the proteinaceous effector ToxA and another eight predicted effectors in Ptr. This highlights the effectiveness of more targeted sampling techniques and experimental designs and addresses the need for their application in sampling symptomatic regions to enable better capture of fungal gene expression *in planta* during infection.

Based on the number of RNA-sequencing reads that aligned to the Ptr genome, our results were in consensus with early and high-level expression of *ToxA* and other predicted NE genes. The detection of Ptr putative effectors and *ToxA* gene expression extends the findings of recent studies that detected the expression of *ToxA* 8 h after Ptr inoculation (Andersen *et al.*, 2021) and the secretion of Ptr effector molecules ahead of the advancing mycelia triggering wheat programmed cell death (Friesen and Faris, 2021). A higher number of Ptr transcript reads were detected in the MR cultivar’s AGR as compared with the SVS cultivar. The reason for this is unclear and requires further investigation. In the MR cultivar, there were two other predicted proteinaceous effectors that had the same or more reads compared with *ToxA*. Although our molecular data are not spatially resolved to the single cell level, the tissue sectioning of the AGR has provided valuable new insights into Ptr–wheat molecular changes and the extent of green tissue loss after disease disruption.

### Conclusions

The work presented here draws on hallmarks of defence presenting new insights into cellular stress and AGR priming ahead of the pathogen invasion front. Necrotrophic fungal pathogens have a complex mode of host manipulation, and our data confirm this for Ptr–wheat. The SVS wheat shows a heightened defence response that is tightly controlled by the pathogen indicating that the healthy cells surrounding necrotic lesions are ‘unaware’ of upcoming spore release and secondary invasion. Our targeted tissue sampling technique in combination with synchrotron microscopy provides the basis for more targeted tissue analysis. Further spatial resolution of infected tissue into various infection zones will alleviate the dilution of key processes essential for recovery. The technique applied to genetically diverse hosts paves the way to characterize complex traits pertaining to disease tolerance, a ripe area of exploration for the future. This in turn gives us the capability to modulate infection and not rely heavily on chemical treatments. Future studies may harness the benefits of single-cell sequencing and availability of genomic sequences in this context which would further enhance our knowledge of the wave of pathogen produced toxins and corresponding changes in plant cell molecular networks.

## Supplementary data

The following supplementary data are available at *JXB* online.

Fig. S1. Optimization of the concentration of fungicide application to suppress disease and minimize adverse impacts of fungicide on the leaves.

Fig. S2. Count of RNA sequenced reads aligned to the wheat genome for each sample, susceptible wheat (cv Scout) and moderately resistant wheat (cv Magenta).

Fig. S3. Principal component analysis of sample normalized gene expression for wheat cultivars (Scout and Magenta) infected with Ptr and treated with fungicide (Scout) with samples collected at 3, 6, and 8 dpi.

Fig S4. High-resolution elemental maps generated for representative wheat leaves infected with Ptr and harvested 3 d post-inoculation and imaged.

Fig S5. Semi-quantitative Ca images for the remainder of three paired replicates for SVS and MR wheat infected with Ptr.

Fig S6. Sample gene expression (log_2_) for NBR-ARC, TraesCS2D02G573600, EDTS5, TraesCS4B02G264500, and two cysteine/histidine-rich DC1 domain-containing genes, TraesCSU02G176200 and TraesCS2B02G521300.

Fig S7. Ptr isolate *ToxA* region (CM025800.1: 1 691 000–1 693 200 bp) with Ptr RNA read alignments.

Dataset S1. Randomized block designs generated using DiGGer v1.0.5.

Dataset S2. Significantly differentially expressed genes based on RNA sample comparisons.

Dataset S3. Significant GO terms enriched by significantly differentially expressed genes.

erad183_suppl_Supplementary_Figures_S1-S7

erad183_suppl_Supplementary_Datasets_S1-S3

## Data Availability

The RNA-sequencing data generated by this study have been deposited in the National Centre for Biotechnology Information (NCBI) sequence read archive (SRA) under BioProject PRJNA798111 accession numbers SRR17648775–SRR17648938. The RNA-sequencing data analyses are available in an RStudio v1.3.1093 (RStudio Team 2020) markdown notebook https://github.com/ccdmb/wheat-agr-tanspot.

## References

[CIT0001] Abu-Jamous B , Kelly S. 2018. Clust: automatic extraction of optimal co-expressed gene clusters from gene expression data. Genome Biology 19, 172. 30359297 10.1186/s13059-018-1536-8 PMC6203272

[CIT0002] Ajigboye OO , Lu C, Murchie EH, Schlatter C, Swart G, Ray RV. 2017. Altered gene expression by sedaxane increases PSII efficiency, photosynthesis and growth and improves tolerance to drought in wheat seedlings. Pesticide Biochemistry and Physiology 137, 49–61. 28364804 10.1016/j.pestbp.2016.09.008

[CIT0003] Ali S , Ganai BA, Kamili AN, et al. 2018. Pathogenesis-related proteins and peptides as promising tools for engineering plants with multiple stress tolerance. Microbiological Research 21, 29–37. 10.1016/j.micres.2018.04.008 29853166

[CIT0004] Ali S , Gurung S, Adhikari TB. 2010. Identification and characterization of novel isolates of *Pyrenophora tritici-repentis* from Arkansas. Plant Disease 94, 229–235. 30754257 10.1094/PDIS-94-2-0229

[CIT0005] Andersen EJ , Nepal MP, Ali S. 2021. Necrotrophic fungus *Pyrenophora tritici-repentis* triggers expression of multiple resistance components in resistant and susceptible wheat cultivars. The Plant Pathology Journal 37, 99–114. 33866753 10.5423/PPJ.OA.06.2020.0109 PMC8053848

[CIT0006] Andrews S. 2011. FastQC. http://www.bioinformatics.babraham.ac.uk/projects/fastqc/.

[CIT0007] Bastiaans L. 1991. Ratio between virtual and visual lesion size as a measure to describe reduction in leaf photosynthesis of rice due to leaf blast. Phytopathology 81, 611–615.

[CIT0008] Benjamini Y , Hochberg Y. 1995. Controlling the false discovery rate – a practical and powerful approach to multiple testing. Journal of the Royal Statistical Society: Series B (Methodological) 57, 289–300.

[CIT0009] Bi G , Su M, Li N, et al. 2021. The ZAR1 resistosome is a calcium-permeable channel triggering plant immune signaling. Cell 184, 3528–3541.e12. 33984278 10.1016/j.cell.2021.05.003

[CIT0010] Ciuffetti LM , Manning VA, Pandelova I, Betts MF, Martinez JP. 2010. Host-selective toxins, Ptr ToxA and Ptr ToxB, as necrotrophic effectors in the *Pyrenophora tritici-repentis*–wheat interaction. New Phytologist 187, 911–919. 20646221 10.1111/j.1469-8137.2010.03362.x

[CIT0011] Ciuffetti LM , Manning VA, Pandelova I, Faris JD, Friesen TL, Strelkov SE, Weber GL, Goodwin SB, Wolpert TJ, Figueroa M. 2014. *Pyrenophora tritici-repentis*: A plant pathogenic fungus with global impact. In: Dean RA, Lichens-Park A, Kole C, eds. Genomics of plant-associated fungi: monocot pathogens. Berlin, Heidelberg: Springer, 1–39.

[CIT0012] Conrath U. 2006. Systemic acquired resistance. Plant Signaling & Behavior 1, 179–184. 19521483 10.4161/psb.1.4.3221 PMC2634024

[CIT0013] Coombes N. 2008. DiGGer. http://nswdpibiom.org/austatgen/software/.

[CIT0014] Dobin A , Davis CA, Schlesinger F, Drenkow J, Zaleski C, Jha S, Batut P, Chaisson M, Gingeras TR. 2013. STAR: ultrafast universal RNA-seq aligner. Bioinformatics 29, 15–21. 23104886 10.1093/bioinformatics/bts635 PMC3530905

[CIT0015] Dushnicky LG , Ballance GM, Sumner MJ, MacGregor AW. 2009. Penetration and infection of susceptible and resistant wheat cultivars by necrosis toxin-producing isolate of *Pyrenophora tritici-repentis*. Canadian Journal of Plant Pathology 18, 392–402.

[CIT0016] Ephrath JE , Shteinberg D, Drieshpoun J, Dinoor A, Marani A. 1989. *Alternaria alternata* in cotton (*Gossypium hirsutum*) cv. Acala: Effects on gas exchange, yield components and yield accumulation. Netherlands Journal of Plant Pathology 95, 157–166.

[CIT0017] Faris JD , Friesen TL. 2020. Plant genes hijacked by necrotrophic fungal pathogens. Current Opinion in Plant Biology 56, 74–80. 32492572 10.1016/j.pbi.2020.04.003

[CIT0018] Faris JD , Liu Z, Xu SS. 2013. Genetics of tan spot resistance in wheat. Theoretical and Applied Genetics 126, 2197–2217. 23884599 10.1007/s00122-013-2157-y

[CIT0019] Faris JD , Zhang Z, Lu H, et al. 2010. A unique wheat disease resistance-like gene governs effector-triggered susceptibility to necrotrophic pathogens. Proceedings of the National Academy of Sciences, USA 107, 13544–13549. 10.1073/pnas.1004090107 PMC2922177 20624958

[CIT0020] Förderer A , Li E, Lawson AW, et al. 2022. A wheat resistosome defines common principles of immune receptor channels. Nature 610, 532–539. 36163289 10.1038/s41586-022-05231-w PMC9581773

[CIT0021] Freeman BC , Beattie GA. 2008. An overview of plant defenses against pathogens and herbivores. The Plant Health Instructor, doi: 10.1094/PHI-I-2008-0226-01.

[CIT0022] Friesen TL , Faris JD. 2021. Characterization of effector-target interactions in necrotrophic pathosystems reveals trends and variation in host manipulation. Annual Review of Phytopathology 59, 77–98. 10.1146/annurev-phyto-120320-012807 33909478

[CIT0023] Howard DL , de Jonge MD, Afshar N, et al. 2020. The XFM beamline at the Australian Synchrotron. Journal of Synchrotron Radiation 27, 1447–1458. 32876622 10.1107/S1600577520010152

[CIT0024] Howe KL , Contreras-Moreira B, De Silva N, et al. 2020. Ensembl Genomes 2020—enabling non-vertebrate genomic research. Nucleic Acids Research 48, D689–D695. 31598706 10.1093/nar/gkz890 PMC6943047

[CIT0025] Hurgobin B , Lewsey MG. 2022. Applications of cell- and tissue-specific ’omics to improve plant productivity. Emerging Topics in Life Sciences 6, 163–173. 35293572 10.1042/ETLS20210286 PMC9023014

[CIT0026] Hwang IS , Choi DS, Kim NH, Kim DS, Hwang BK. 2014. The pepper cysteine/histidine-rich DC1 domain protein CaDC1 binds both RNA and DNA and is required for plant cell death and defense response. New Phytologist 201, 518–530. 24117868 10.1111/nph.12521

[CIT0027] International Wheat Genome Sequencing Consortium. 2014. A chromosome-based draft sequence of the hexaploid bread wheat (*Triticum aestivum*) genome. Science 345, 1251788. 25035500 10.1126/science.1251788

[CIT0028] Isono K , Shimizu M, Yoshimoto K, Niwa Y, Satoh K, Yokota A, Kobayashi H. 1997. Leaf-specifically expressed genes for polypeptides destined for chloroplasts with domains of sigma70 factors of bacterial RNA polymerases in *Arabidopsis thaliana*. Proceedings of the National Academy of Sciences, USA 94, 14948–14953. 10.1073/pnas.94.26.14948 PMC25143 9405719

[CIT0029] Khambatta K , Hollings A, Sauzier G, et al. 2021. “Wax on, wax off”: in vivo imaging of plant physiology and disease with Fourier transform infrared reflectance microspectroscopy. Advanced Science 8, 2101902. 34338438 10.1002/advs.202101902 PMC8498906

[CIT0030] Lamari L , Bernier CC. 1989a. Evaluation of wheat lines and cultivars to tan spot [*Pyrenophora tritici-repentis*] based on lesion type. Canadian Journal of Plant Pathology–Revue Canadienne De Phytopathologie 11, 49–56.

[CIT0031] Lamari L , Bernier CC. 1989b. Virulence of isolates of *Pyrenophora tritici-repentis* on 11 wheat cultivars and cytology of the differential host reactions. Canadian Journal of Plant Pathology 11, 284–290.

[CIT0032] Liew LC , Narsai R, Wang Y, Berkowitz O, Whelan J, Lewsey MG. 2020. Temporal tissue-specific regulation of transcriptomes during barley (*Hordeum vulgare*) seed germination. The Plant Journal 101, 700–715. 31628689 10.1111/tpj.14574

[CIT0033] Lightfoot DJ , Able AJ. 2010. Growth of *Pyrenophora teres* in planta during barley net blotch disease. Australasian Plant Pathology 39, 499–507.

[CIT0034] Liu Z , Friesen TL, Ling H, Meinhardt SW, Oliver RP, Rasmussen JB, Faris JD. 2006. The Tsn1–ToxA interaction in the wheat–*Stagonospora nodorum* pathosystem parallels that of the wheat–tan spot system. Genome 49, 1265–1273. 17213908 10.1139/g06-088

[CIT0035] Love MI , Huber W, Anders S. 2014. Moderated estimation of fold change and dispersion for RNA-seq data with DESeq2. Genome Biology 15, 550. 25516281 10.1186/s13059-014-0550-8 PMC4302049

[CIT0036] Manning VA , Chu AL, Steeves JE, Wolpert TJ, Ciuffetti LM. 2009. A host-selective toxin of *Pyrenophora tritici-repentis*, Ptr ToxA, induces photosystem changes and reactive oxygen species accumulation in sensitive wheat. Molecular Plant-Microbe Interactions 22, 665–676. 19445591 10.1094/MPMI-22-6-0665

[CIT0037] Manning VA , Pandelova I, Dhillon B, et al. 2013. Comparative genomics of a plant-pathogenic fungus, *Pyrenophora tritici-repentis*, reveals transduplication and the impact of repeat elements on pathogenicity and population divergence. G3: Genes, Genomes, Genetics 3, 41–63. 23316438 10.1534/g3.112.004044 PMC3538342

[CIT0038] Martin M. 2011. Cutadapt removes adapter sequences from high-throughput sequencing reads. EMBnet.journal 17, doi: 10.14806/ej.17.1.200.

[CIT0039] Mengiste T. 2012. Plant immunity to necrotrophs. Annual Review of Phytopathology 50, 267–294. 10.1146/annurev-phyto-081211-172955 22726121

[CIT0040] Mitchell AL , Attwood TK, Babbitt PC, et al. 2019. InterPro in 2019: improving coverage, classification and access to protein sequence annotations. Nucleic Acids Research 47, D351–D360. 30398656 10.1093/nar/gky1100 PMC6323941

[CIT0041] Moffat CS , See PT, Oliver RP. 2014. Generation of a ToxA knockout strain of the wheat tan spot pathogen *Pyrenophora tritici-repentis*. Molecular Plant Pathology 15, 918–926. 24831982 10.1111/mpp.12154 PMC6638721

[CIT0042] Moolhuijzen P , See PT, Hane JK, Shi G, Liu Z, Oliver RP, Moffat CS. 2018. Comparative genomics of the wheat fungal pathogen *Pyrenophora tritici-repentis* reveals chromosomal variations and genome plasticity. BMC Genomics 19, 279. 29685100 10.1186/s12864-018-4680-3 PMC5913888

[CIT0043] Naim F , Khambatta K, Sanglard LMVP, Sauzier G, Reinhardt J, Paterson DJ, Zerihun A, Hackett MJ, Mark RG. 2021. Synchrotron X-ray fluorescence microscopy-enabled elemental mapping illuminates the “battle for nutrients” between plant and pathogen. Journal of Experimental Botany 72, 2757–2768. 33439999 10.1093/jxb/erab005 PMC8006550

[CIT0044] Oliver R , Osbourn A. 1995. Molecular dissection of fungal phytopathogenicity. Microbiology 141, 1–9. 7894700 10.1099/00221287-141-1-1

[CIT0045] van Ooijen G , Mayr G, Kasiem MM, Albrecht M, Cornelissen BJ, Takken FL. 2008. Structure-function analysis of the NB-ARC domain of plant disease resistance proteins. Journal of Experimental Botany 59, 1383–1397. 18390848 10.1093/jxb/ern045

[CIT0046] Paterson D , Jonge MD, Howard DL, Lewis W, McKinlay J, Starritt A, Kusel M, Ryan CG, Kirkham R, et al. 2011. The X-ray fluorescence microscopy beamline at the Australian Synchrotron. American Institute of Physics Conference Proceedings 1365, 219–222.

[CIT0047] Quevillon E , Silventoinen V, Pillai S, Harte N, Mulder N, Apweiler R, Lopez R. 2005. InterProScan: protein domains identifier. Nucleic Acids Research 33, W116–W120. 15980438 10.1093/nar/gki442 PMC1160203

[CIT0048] Rawlings ND , Barrett AJ, Thomas PD, Huang X, Bateman A, Finn RD. 2018. The MEROPS database of proteolytic enzymes, their substrates and inhibitors in 2017 and a comparison with peptidases in the PANTHER database. Nucleic Acids Research 46, D624–D632. 29145643 10.1093/nar/gkx1134 PMC5753285

[CIT0049] Rawlinson C , See PT, Moolhuijzen P, Li H, Moffat CS, Chooi YH, Oliver RP. 2019. The identification and deletion of the polyketide synthase-nonribosomal peptide synthase gene responsible for the production of the phytotoxic triticone A/B in the wheat fungal pathogen *Pyrenophora tritici-repentis*. Environmental Microbiology 21, 4875–4886. 31698543 10.1111/1462-2920.14854 PMC6915911

[CIT0050] RStudio Team. 2020. RStudio: Integrated Development Environment for R. 1.3.1093. http://www.rstudio.com/.

[CIT0051] Ryan CG. 2000. Quantitative trace element imaging using PIXE and the nuclear microprobe. International Journal of Imaging Systems and Technology 11, 219–230.

[CIT0052] Salguero-Linares J , Serrano I, Ruiz-Solani N, Salas-Gómez M, Phukan UJ, González VM, Bernardo-Faura M, Valls M, Rengel D, Coll NS. 2022. Robust transcriptional indicators of immune cell death revealed by spatiotemporal transcriptome analyses. Molecular Plant 15, 1059–1075. 35502144 10.1016/j.molp.2022.04.010

[CIT0053] Schindelin J , Rueden CT, Hiner MC, Eliceiri KW. 2015. The ImageJ ecosystem: An open platform for biomedical image analysis. Molecular Reproduction and Development 82, 518–529. 26153368 10.1002/mrd.22489 PMC5428984

[CIT0054] See PT , Marathamuthu KA, Iagallo EM, Oliver RP, Moffat CS. 2018. Evaluating the importance of the tan spot ToxA–Tsn1 interaction in Australian wheat varieties. Plant Pathology 67, 1066–1075.

[CIT0055] Serrago RA. 2009. Foliar diseases affect the eco-physiological attributes linked with yield and biomass in wheat (*Triticum aestivum* L.). European Journal of Agronomy 31, 195–203.

[CIT0056] Shao Z-Q , Xue J-Y, Wang Q, Wang B, Chen J-Q. 2019. Revisiting the origin of plant NBS-LRR genes. Trends in Plant Science 24, 9–12. 30446304 10.1016/j.tplants.2018.10.015

[CIT0057] Shi G , Zhang Z, Friesen TL, Bansal U, Cloutier S, Wicker T, Rasmussen JB, Faris JD. 2016. Marker development, saturation mapping, and high-resolution mapping of the *Septoria nodorum* blotch susceptibility gene Snn3-B1 in wheat. Molecular Genetics and Genomics 291, 107–119. 26187026 10.1007/s00438-015-1091-x

[CIT0058] Smedley D , Haider S, Durinck S, et al. 2015. The BioMart community portal: an innovative alternative to large, centralized data repositories. Nucleic Acids Research 43, W589–W598. 25897122 10.1093/nar/gkv350 PMC4489294

[CIT0059] Tan K-C , Oliver RP, Solomon PS, Moffat CS. 2010. Proteinaceous necrotrophic effectors in fungal virulence. Functional Plant Biology 37, 907–912.

[CIT0060] Tian T , Liu Y, Yan H, You Q, Yi X, Du Z, Xu W, Su Z. 2017. agriGO v2.0: a GO analysis toolkit for the agricultural community, 2017 update. Nucleic Acids Research 45, W122–W129. 28472432 10.1093/nar/gkx382 PMC5793732

[CIT0061] Verwaaijen B , Wibberg D, Winkler A, Zrenner R, Bednarz H, Niehaus K, Grosch R, Pühler A, Schlüter A. 2019. A comprehensive analysis of the *Lactuca sativa*, L. transcriptome during different stages of the compatible interaction with *Rhizoctonia solani*. Scientific Reports 9, 7221. 31076623 10.1038/s41598-019-43706-5 PMC6510776

[CIT0062] Wang X , Jiang N, Liu J, Liu W, Wang G-L. 2014. The role of effectors and host immunity in plant–necrotrophic fungal interactions. Virulence 5, 722–732. 25513773 10.4161/viru.29798 PMC4189878

[CIT0063] Whelan HG , Gaunt RE. 1990. Yield loss: disease relationships in barley crops with different yield potentials. Proceedings of the Forty Third New Zealand Weed and Pest Control Conference, Dunedin, August 14–19, 1990. Palmerston North, New Zealand: Weed and Pest Control Society, 159–162.

[CIT0064] Yuan P , Jauregui E, Du L, Tanaka K, Poovaiah BW. 2017. Calcium signatures and signaling events orchestrate plant–microbe interactions. Current Opinion in Plant Biology 38, 173–183. 28692858 10.1016/j.pbi.2017.06.003

