# Supplementary material for: Spatiotemporal patterns of wheat response to Pyrenophora tritici-repentis in asymptomatic regions revealed by transcriptomic and X-ray fluorescence microscopy analyses
Source: J Exp Bot. 2023 May 18;74(15):4707–20. doi: 10.1093/jxb/erad183 (PMC10433925; doi:10.1093/jxb/erad183)
Supplement: erad183_suppl_Supplementary_Figures_S1-S7 [file erad183_suppl_supplementary_figures_s1-s7.pdf]

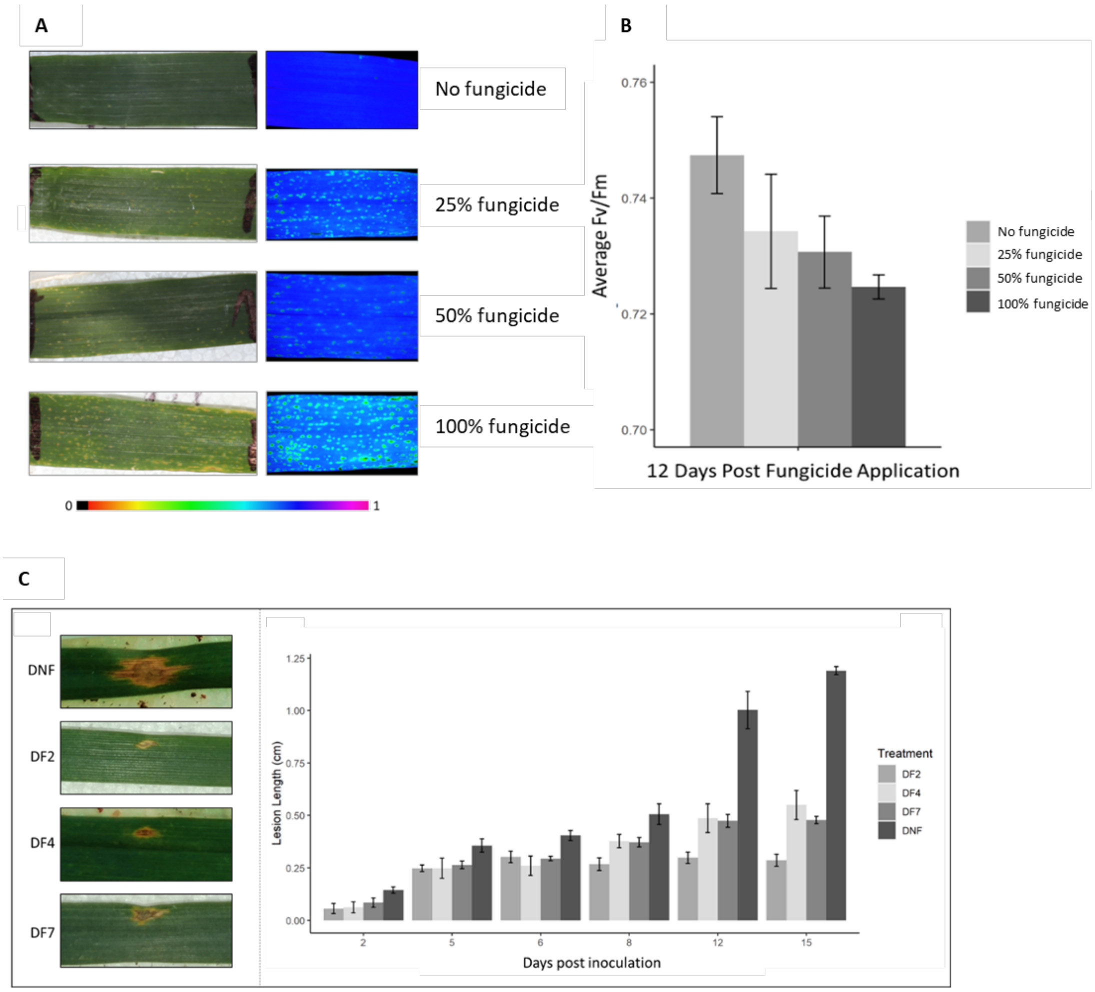

Supplementary Fig. S1. Optimisation of the concentration of fungicide application to suppress disease and minimise adverse impacts of fungicide on the leaves. **(A-B)** wheat leaves treated with 0, 25%, 50% and 100% of the recommended label rate of Prosara® and monitored for 12 days post application (dpfa). **(A)** Representative leaves displaying speckling 12 dpfa, left – digital camera image, right – Chlorophyl fluorescence heatmap of Fv/Fm. **(B)** Average Fv/Fm of uninoculated leaves sprayed with various rates of fungicide (0, 25, 50 and 100%), 12 dpfa (*n* = 5). The leaves treated with higher concentration of fungicide showed significantly more yellowing and therefore reduction in photosynthetic capacity. **(C)** Left panel: Images of representative wheat (cv Scout) leaves inoculated with Ptr and exposed to 25% diluted fungicide at different time points post inoculation, right panel: the corresponding lesion length. Average lesion length measured for up to 14 days post inoculation (dpi) (*n* = 5). Abbreviations:

DNF = disease and no fungicide, DF2 = disease and fungicide applied at 2 dpi, DF4 = disease and fungicide at 4 dpi and DF7 = disease and fungicide applied at 7 dpi), photographed 14 dpi. Error bars are standard error of the mean.

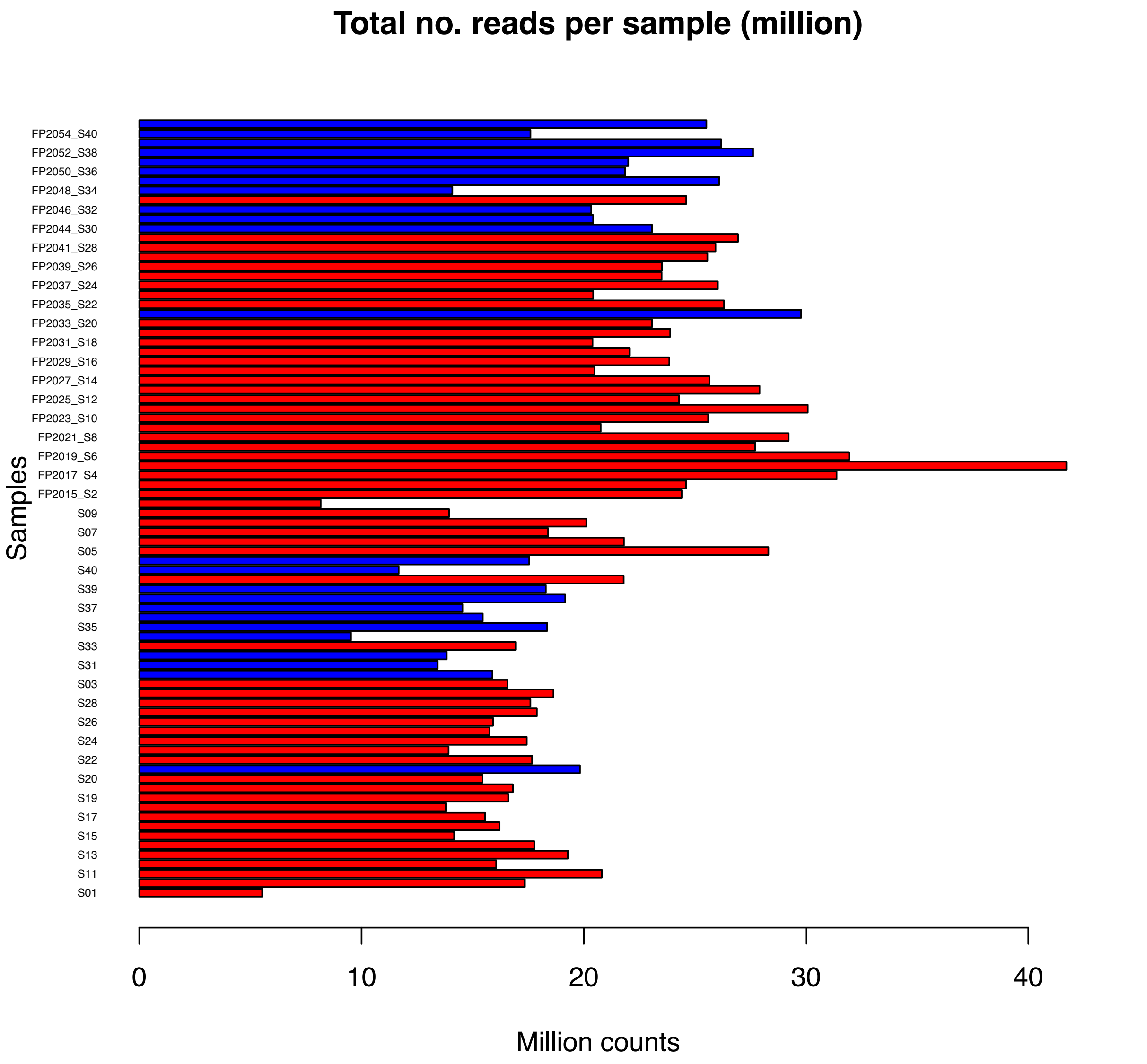

Supplementary Fig. S2. Count of RNA sequenced reads aligned to the wheat genome for each sample, susceptible wheat (cv Scout) in red and moderately resistant wheat (cv Magenta) in blue.

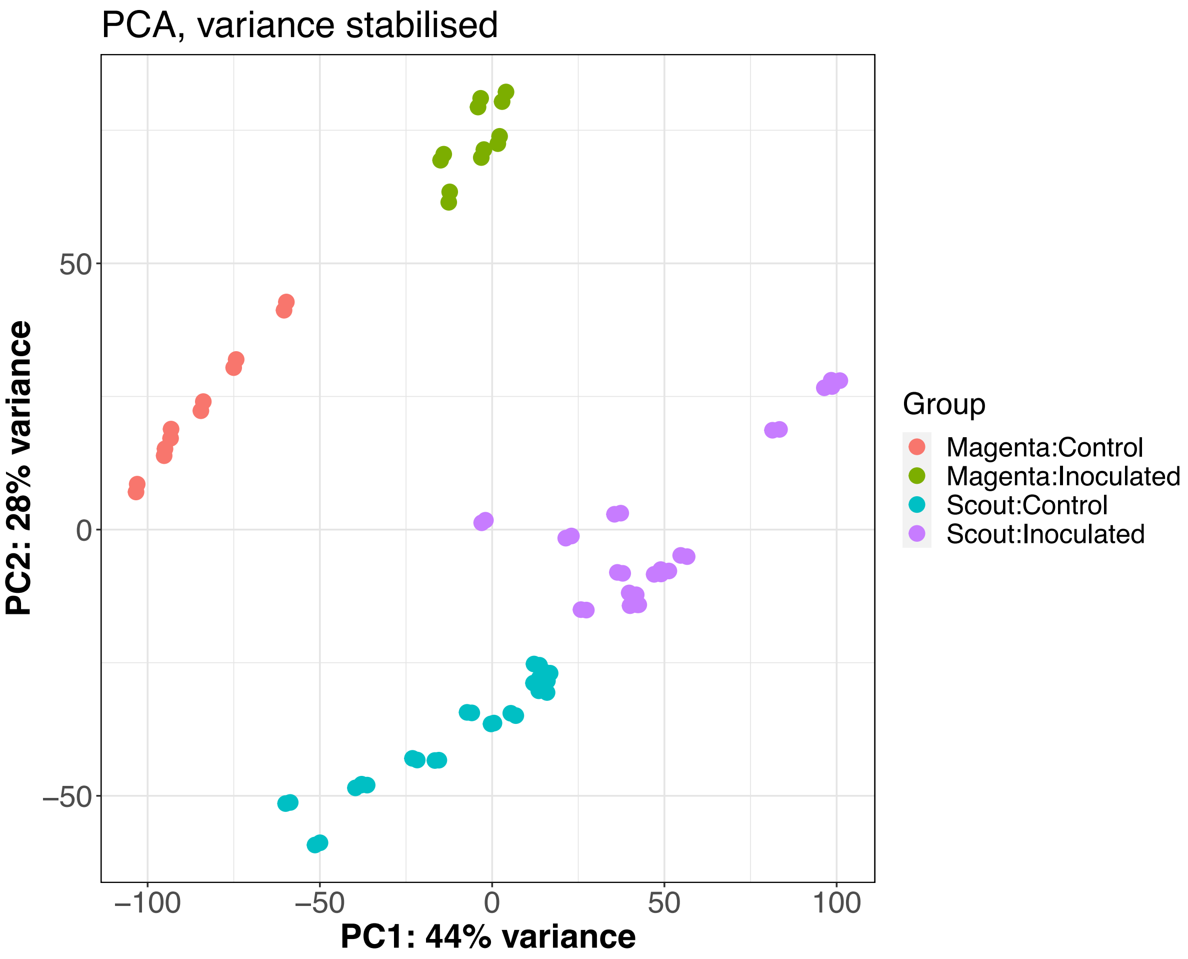

Supplementary Fig. S3. Principle component analysis of sample normalised gene expression for wheat cultivars (Scout and Magenta) infected with Ptr and treated with fungicide (Scout) and samples collected at 3, 6 and 8 dpi.

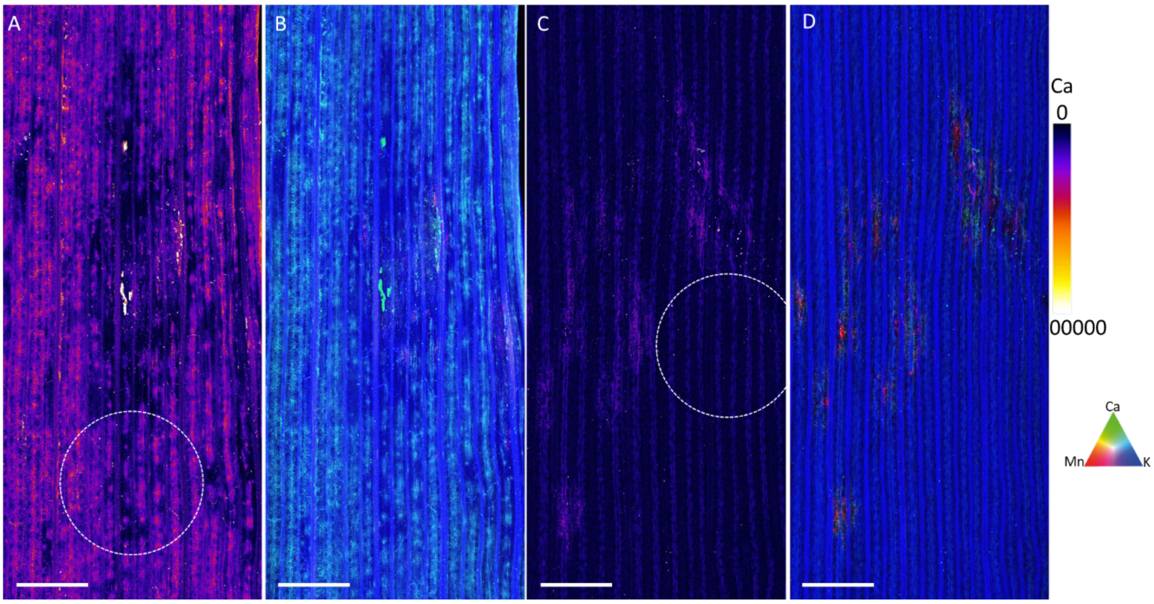

Supplementary Fig S4. High-resolution elemental maps generated for representative wheat leaves infected with Ptr and harvested 3 days post inoculation and imaged. XFM Ca maps for (A) susceptible (SVS) and (C) moderately resistant (MR) The corresponding tricolour overlay of element maps for Mn (red), Ca (green) and K (blue) shown in (B) and (D), respectively. The AGR is indicated by the white dashed rectangle. Each map is false coloured to help visualise the patterns of elemental re-distribution and brightness and contrast were kept consistent for each element. Scale bar = 1 mm, and elemental maps are expressed in units of ng cm^{-2}.

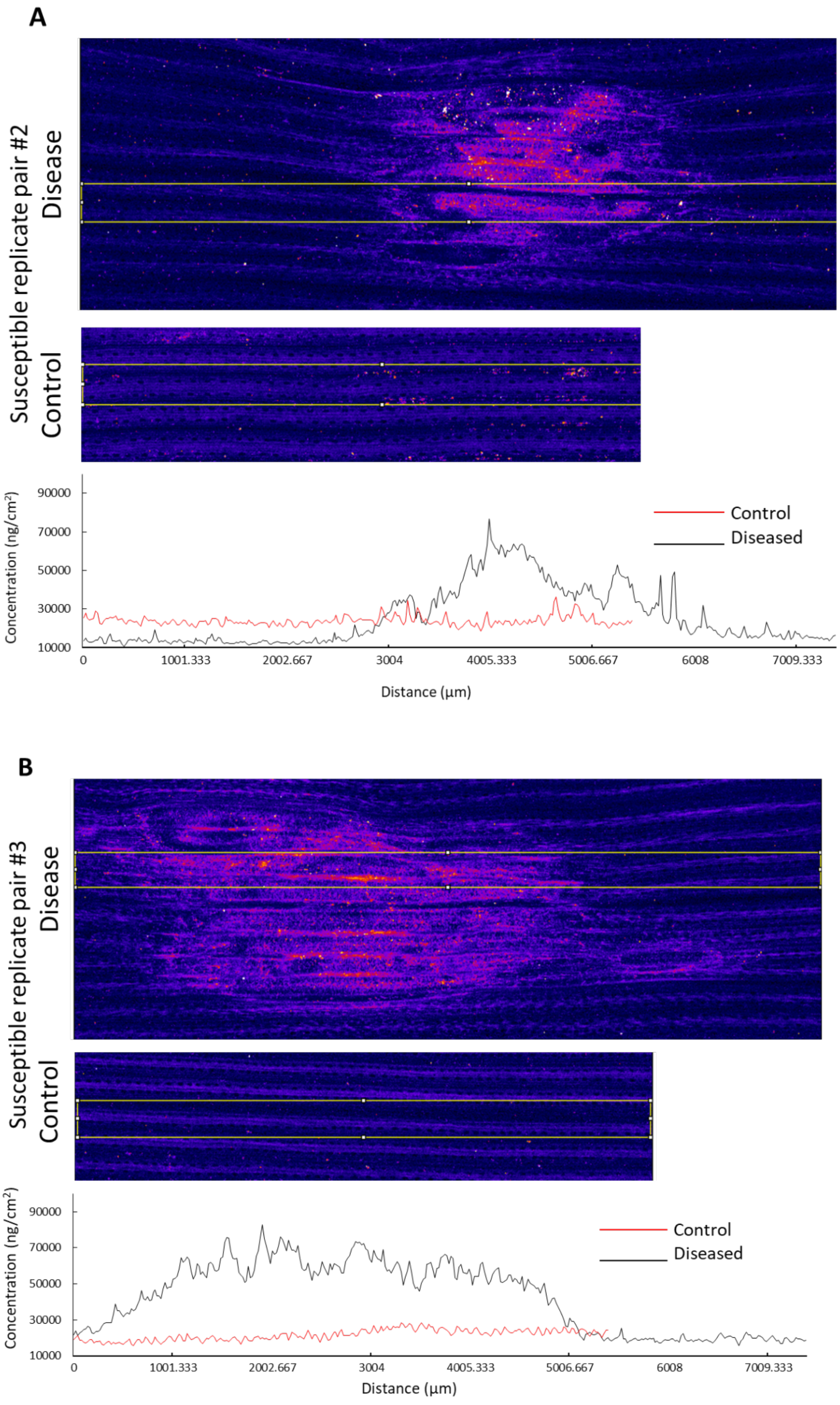
A
Susceptible replicate pair #2
Disease
Control
Concentration (ng/cm²)
90000
70000
50000
30000
10000
0
1001.333
2002.667
3004
4005.333
5006.667
6008
7009.333
Distance (µm)
Control
Diseased
B
Susceptible replicate pair #3
Disease
Control
Concentration (ng/cm²)
90000
70000
50000
30000
10000
0
1001.333
2002.667
3004
4005.333
5006.667
6008
7009.333
Distance (µm)
Control
Diseased

C

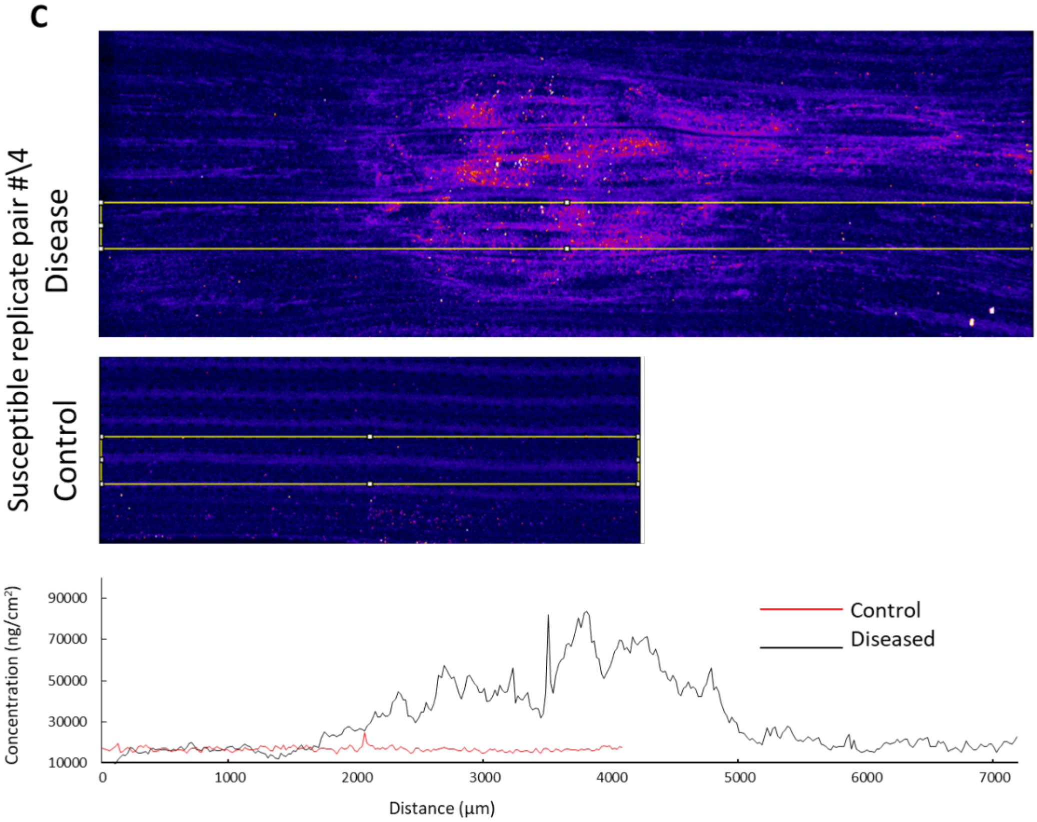

Susceptible replicate pair #\4
Disease
Control
Concentration (ng/cm²)
90000
70000
50000
30000
10000
0
1000
2000
3000
4000
5000
6000
7000
Distance (µm)
Control
Diseased

D

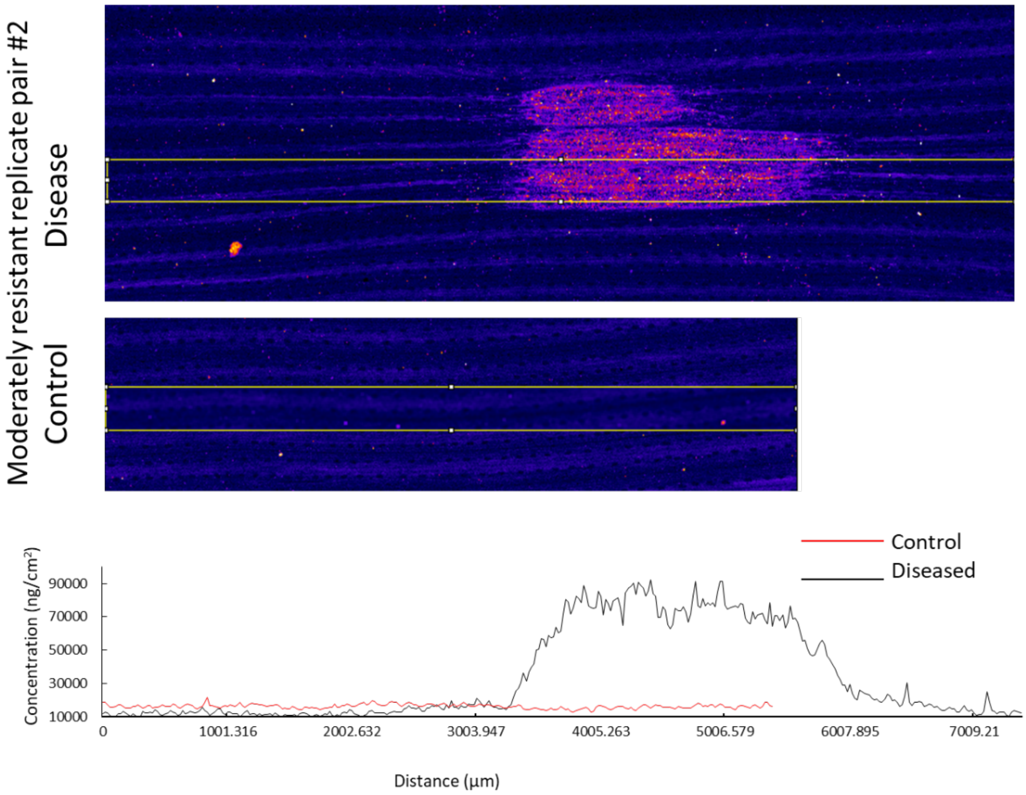

Moderately resistant replicate pair #2
Disease
Control
Concentration (ng/cm²)
90000
70000
50000
30000
10000
0
1001.316
2002.632
3003.947
4005.263
5006.579
6007.895
7009.21
Distance (µm)
Control
Diseased

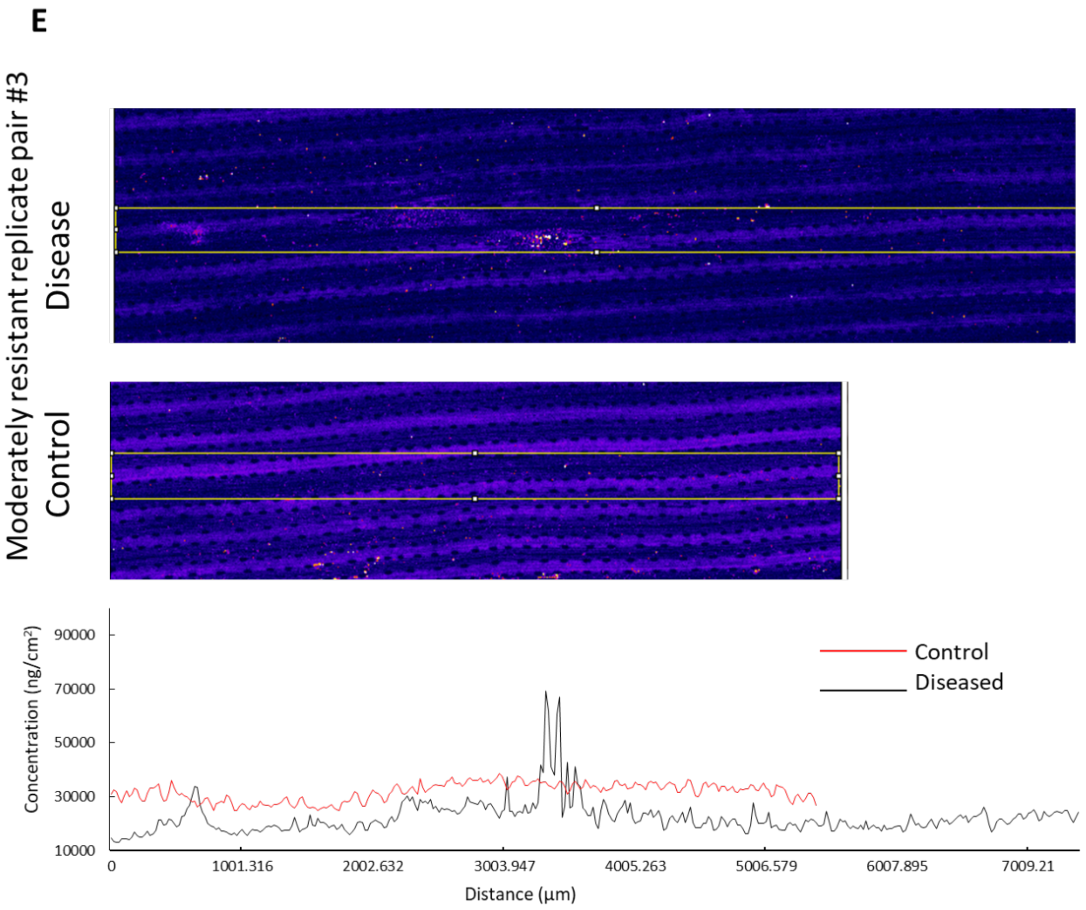
E
Moderately resistant replicate pair #3
Disease
Control
Concentration (ng/cm²)
90000
70000
50000
30000
10000
Control
Diseased
0
1001.316
2002.632
3003.947
4005.263
5006.579
6007.895
7009.21
Distance (μm)

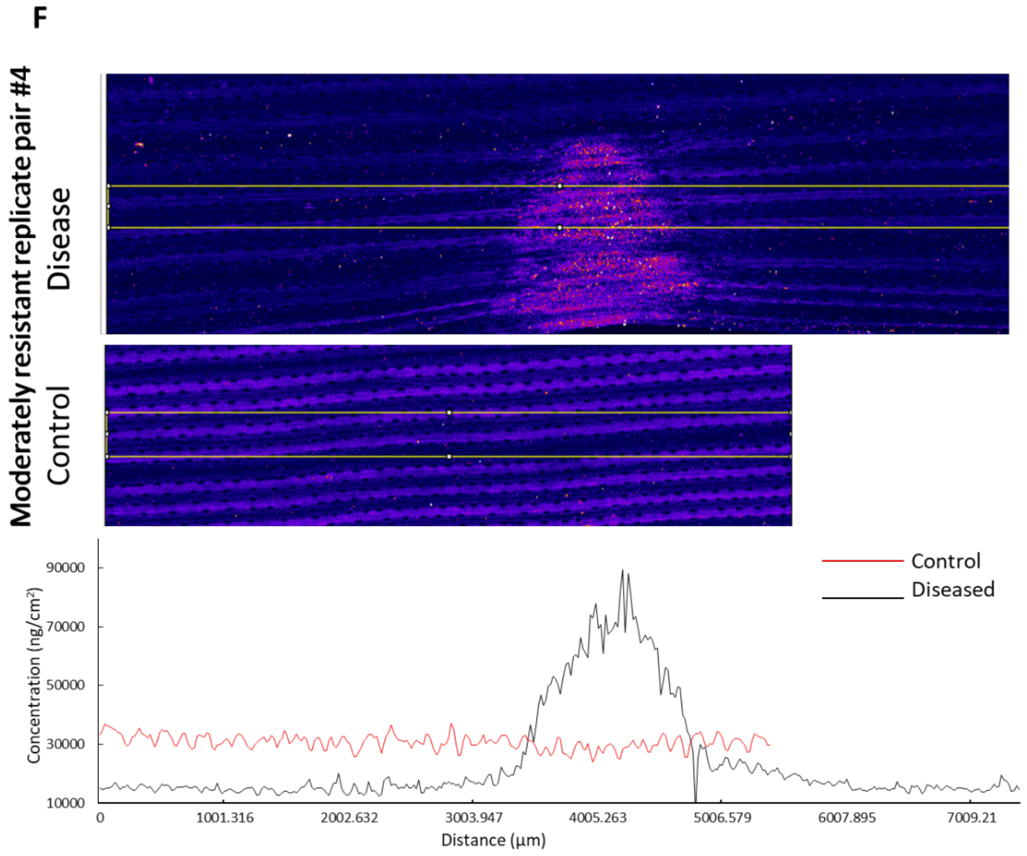
F
Moderately resistant replicate pair #4
Disease
Control
Concentration (ng/cm²)
90000
70000
50000
30000
10000
Control
Diseased
0
1001.316
2002.632
3003.947
4005.263
5006.579
6007.895
7009.21
Distance (μm)

Supplementary Fig S5. Semi-quantitative Ca images for the remainder of 3 paired replicates for SVS and MR wheat infected with Ptr. XFM Ca maps for (A-C) susceptible (SVS) and (D-F) moderately resistant (MR) wheat infected with Ptr and harvested 8 days post inoculation. The Ca maps are false coloured to help visualise the patterns of elemental re-distribution and brightness and contrast were kept consistent for the two cultivars. The concentration of Ca is measured across the leaf indicated by the yellow dashed regions, upper panel is infected leaf section and lower panel is paired control leaf section. Replicate pair 1 data is shown in Figure 2 and due to scan failures, the data for 1 paired replicate for each cultivar was not available.

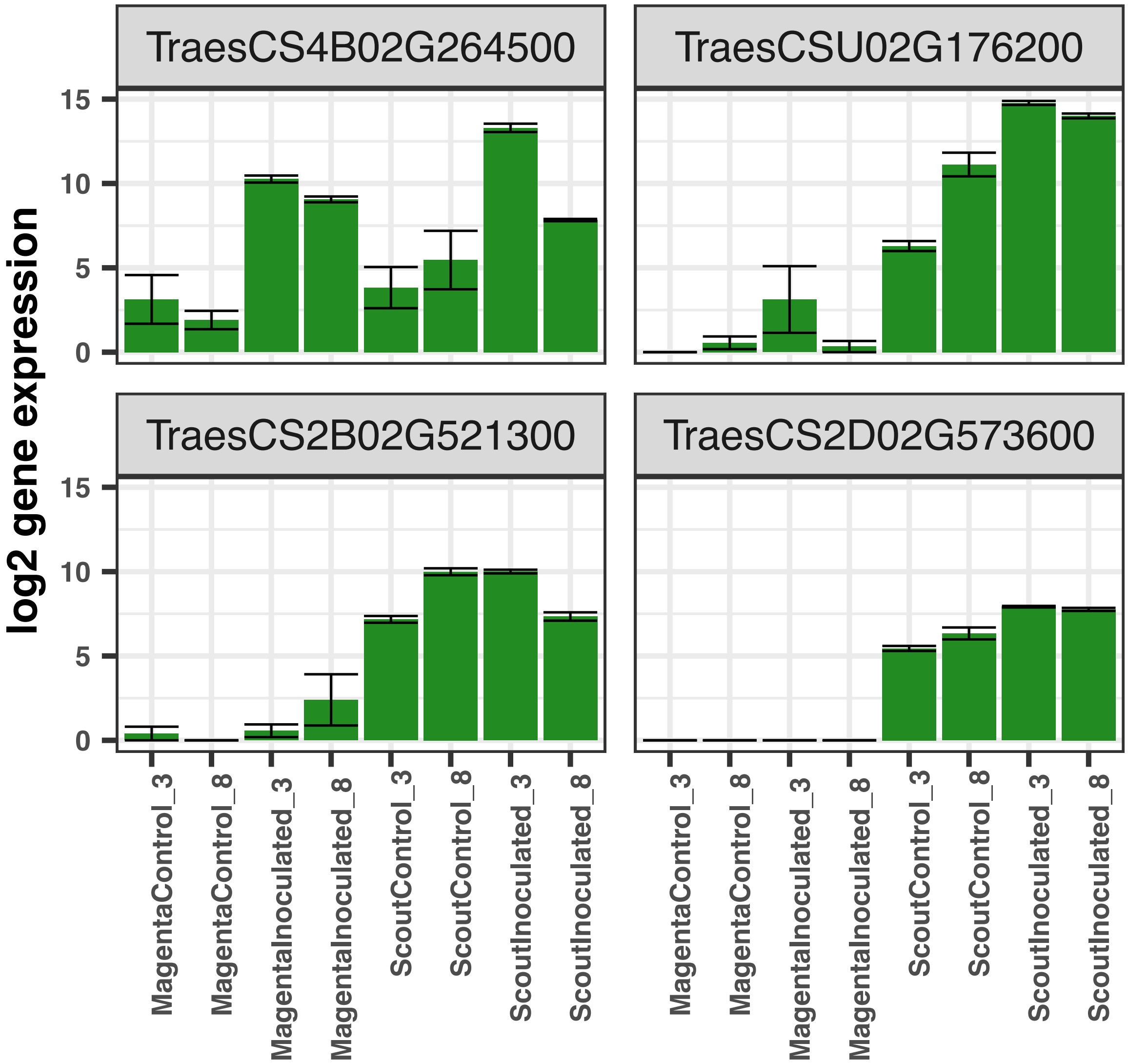

Supplementary Fig S6. Sample gene expression (log_2) for NBR-ARC TraesCS2D02G573600, EDTS5 TraesCS4B02G264500, and two cysteine/histidine-rich DC1 domain containing TraesCSU02G176200 and TraesCS2B02G521300 genes.

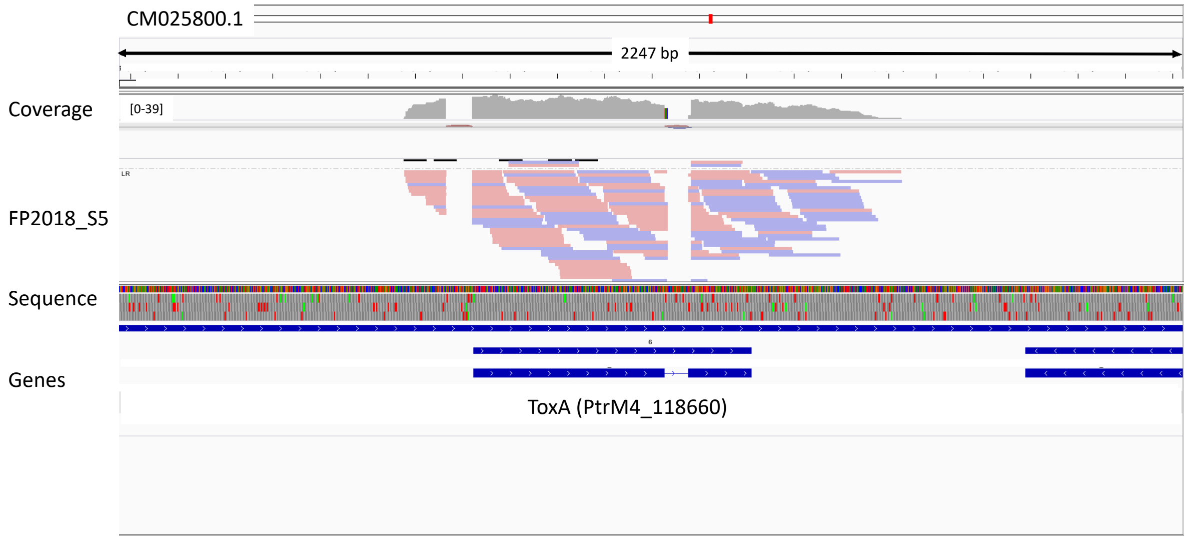

Supplementary Fig S7. Ptr isolate M4 *ToxA* region (CM025800.1:1,691,000-1,693,200 bp) with Ptr RNA read alignments (sample S5). Top to bottom is read coverage (grey), read alignments (pink and purple), genome region forward three frame translation, M4 gene PtrM4_118660 coding regions and exons (blue).
